# Lithium-mediated electrochemical nitrogen reduction: Mechanistic insights to enhance performance

**DOI:** 10.1016/j.isci.2021.103105

**Published:** 2021-09-09

**Authors:** Xiyang Cai, Cehuang Fu, Haldrian Iriawan, Fan Yang, Aiming Wu, Liuxuan Luo, Shuiyun Shen, Guanghua Wei, Yang Shao-Horn, Junliang Zhang

**Affiliations:** 1 Institute of Fuel Cells, School of Mechanical Engineering, Shanghai Jiao Tong University, Shanghai 200240, China; 2 Department of Materials Science and Engineering, Massachusetts Institute of Technology, Cambridge, MA 02139, USA; 3 Department of Materials, Imperial College London, London SW7 5RB, UK; 4 SJTU-Paris Tech Elite Institute of Technology, Shanghai Jiao Tong University, Shanghai 200240, China; 5 Department of Mechanical Engineering, Massachusetts Institute of Technology, Cambridge, MA 02139, USA

**Keywords:** Chemical reaction, Electrochemistry, Chemical synthesis

## Abstract

Green synthesis of ammonia by electrochemical nitrogen reduction reaction (NRR) shows great potential as an alternative to the Haber-Bosch process but is hampered by sluggish production rate and low Faradaic efficiency. Recently, lithium-mediated electrochemical NRR has received renewed attention due to its reproducibility. However, further improvement of the system is restricted by limited recognition of its mechanism. Herein, we demonstrate that lithium-mediated NRR began with electrochemical deposition of lithium, followed by two chemical processes of dinitrogen splitting and protonation to ammonia. Furthermore, we quantified the extent to which the freshly deposited active lithium lost its activity toward NRR due to a parasitic reaction between lithium and electrolyte. A high ammonia yield of 0.410 ± 0.038 μg s^−1^ cm^−2^ geo and Faradaic efficiency of 39.5 ± 1.7% were achieved at 20 mA cm^−2^ geo and 10 mA cm^−2^ geo, respectively, which can be attributed to fresher lithium obtained at high current density.

## Highlights

• Characteristics of nitrogen splitting and protonation processes are clarified
• Parasitic reactions between metallic lithium and tetrahydrofuran are revealed
• Fresh lithium model is employed to rationalize performance dependence on current
• Top-level ammonia yield of 0.410 μg_NH3_ s^−1^ cm^−2^ geo is achieved

## Graphical abstract

**Figure:**
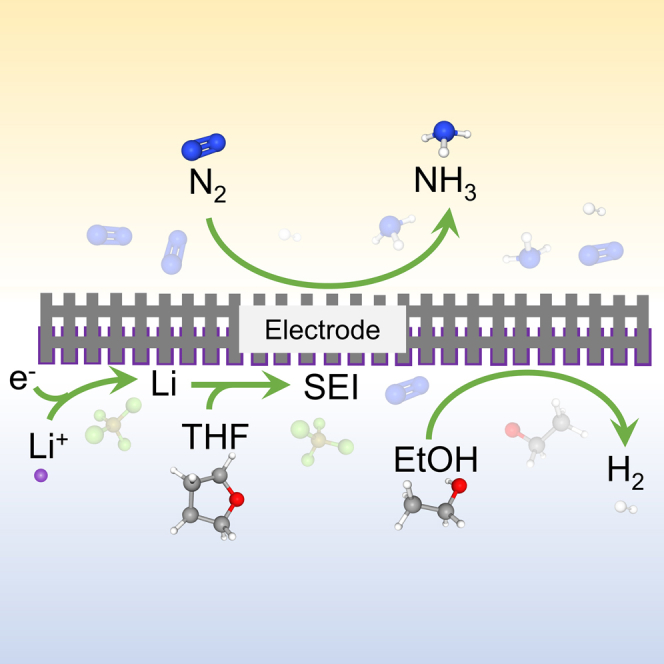

## Introduction

Ammonia (NH_3_) is vital to human society. Regarded as an important feedstock, ammonia is widely used in pharmacy, military, and industry. It is also applied to fertilizer production which makes it possible to secure food supply in the context of continuous population growth (Erisman et al., 2008). Ammonia is mainly produced by the Haber-Bosch process under high temperature and high pressure (300–550°C, 200–350 atm) with continuous feed of hydrogen gas derived from steam reforming (Chen et al., 2018a; Shipman and Symes, 2017), which gives rise to ∼1% of global energy consumption and ∼1% total CO_2_ emission (Soloveichik, 2019; van der Ham et al., 2014). Besides, the extreme reaction condition results in highly centralized production and thus additional cost for long distance transportation of ammonia (Comer et al., 2019). As a promising alternative, ammonia synthesis from electrochemical nitrogen reduction reaction (NRR) can be driven by renewable electrical energy instead of thermal energy (Chen et al., 2018b), enabling ammonia production at ambient conditions, decarbonization and on-site production.

Although electrochemical ammonia synthesis has great promise, the research is still in its early stage. Sluggish splitting of inert N≡N triple bond (Hou et al., 2020; Rostamikia et al., 2019) and competing hydrogen evolution reaction (HER) (Drazevic and Skulason, 2020; Kibsgaard et al., 2019; Singh et al., 2016) are the two main issues faced by researchers. The sheer selectivity challenge against HER, as well as the trace amounts of ammonia measured which could be attributed to nitrogen contaminants (from laboratory glassware (Suryanto et al., 2019), gas feed impurities (Andersen et al., 2019; Cai et al., 2021; Choi et al., 2020b), catalyst preparation (Chen et al., 2020; Liu et al., 2020), Nafion membrane (Andersen et al., 2019; Liu et al., 2020), etc.) rather than genuine N_2_ activation, leads to much contention regarding the fidelity of results in aqueous systems (Andersen et al., 2019; Chen et al., 2020; Choi et al., 2020a, 2020b; Kibsgaard et al., 2019). Following a rigorous testing by Andersen et al. in 2019 via quantitative ^15^N_2_ isotope experiments, the lithium-mediated strategy coupled with nonaqueous electrolytes (such as tetrahydrofuran, THF) is one of the only reliably reproducible systems for ammonia production under ambient conditions (Andersen et al., 2019; Choi et al., 2020b).

First envisioned by the early works of Fichtner et al. in 1931 (Fichter et al., 1930) and Tsuneto's group in 1993 and 1994 (Akira et al., 1994; Tsuneto et al., 1993), the lithium-mediated NRR is generally understood to start with lithium electro-deposition. On the surface of lithium-containing deposits, dinitrogen is activated, followed by the production of ammonia (Akira et al., 1994; Lazouski et al., 2019). In addition, the use of nonaqueous electrolyte constrains access to protons at the catalytic active site, suppressing the parasitic HER. Therefore, active lithium and inert proton source can facilitate N_2_ activation and HER suppression, respectively. To date, lithium-mediated NRR is developing quickly (Andersen et al., 2019, 2020; Lazouski et al., 2019, 2020) and has become an important technological roadmap in the field of electrochemical NRR. However, despite the wide attention raised by researchers and an increasing number of reports (Andersen et al., 2019; Suryanto et al., 2021), the mechanistic understanding on nitrogen reduction in the lithium-mediated process is still in its infancy, which hampers performance enhancement. To unveil the underlying mechanism, the following fundamental but critical questions should be addressed.

### Are the splitting and protonation of dinitrogen electrochemical or chemical processes?

**Figure 1:**
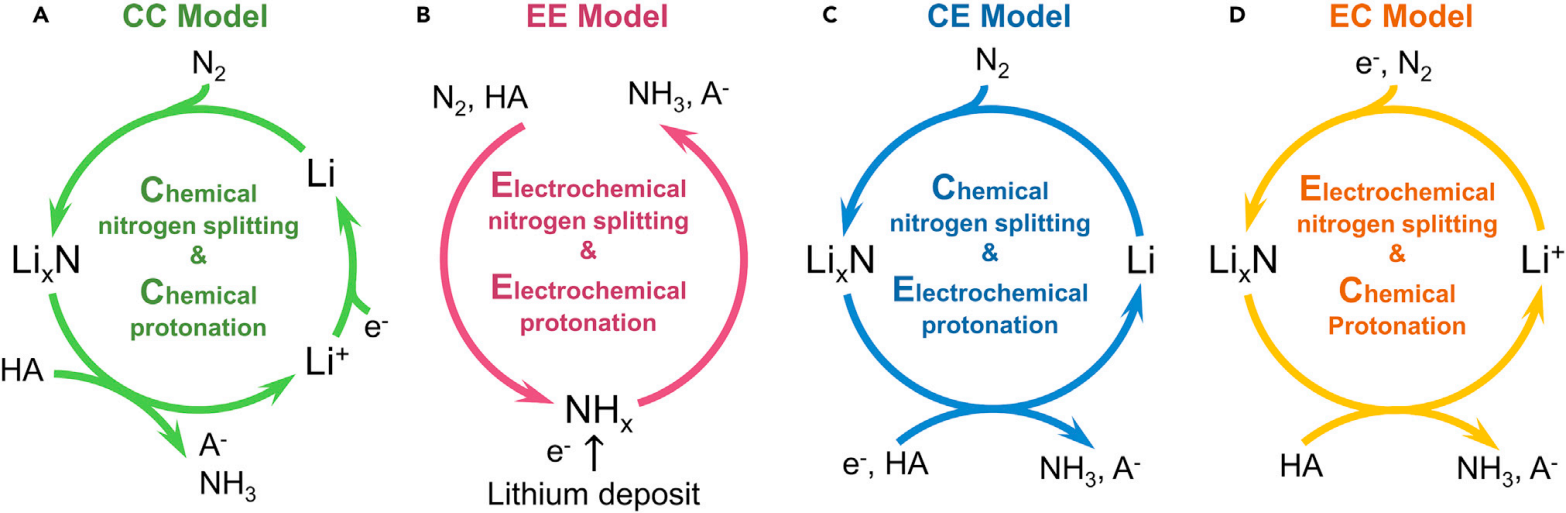
Schematic diagram of lithium-mediated nitrogen reduction reaction Four possible mechanisms of lithium-mediated nitrogen reduction reaction in literature, labeled as CC (Akira et al., 1994; Gao et al., 2020; Lazouski et al., 2019), EE (Schwalbe et al., 2020), CE (Andersen et al., 2020), and EC (Ma et al., 2017; Zhang et al., 2019) model. C and E represent chemical and electrochemical steps, respectively.

Two main constituents of the N_2_ reduction process are (i) the activation/splitting of N_2_ and the (ii) protonation of activated nitrogen species to form ammonia. According to characteristics of N_2_ splitting and protonation, four possible mechanisms can be envisioned (Figure 1). In the chemical N_2_ splitting & chemical protonation model (abbreviated as CC model, Figure 1A), N≡N is split by spontaneous chemical reaction with electrodeposited Li to form lithium nitride. This implies that the activation/splitting of N_2_ occurs by interaction with electrons stored in the electrodeposited metallic lithium. Moreover, the chemical protonation of activated nitrogen (i.e., lithium nitride) to form ammonia is accompanied by the release of Li^+^ into the solution. N_2_ activation only depends on the properties of lithium deposit. Thus, nitrogen reduction/ammonia synthesis can proceed as long as there is residual active lithium deposit on the electrode, even if the current is cutoff.

On the other hand, high energy electrons are also readily available at the Li-plating potential which could in principle reduce incoming N_2_ and protons electrochemically to form ammonia (Figure 1B). In this scheme, a long-living lithium layer could serve as electrocatalysts, assisting coupled proton-electron transfers in a heterogenous catalytic cycle (Schwalbe et al., 2020). As the transfer of protons and electrons are coupled, the dissolution of metallic Li^+^ should not occur. In the case where both N_2_ splitting/activation and protonation are electrochemical processes, dinitrogen splitting and protonation can be manipulated by changing the electrode surface or the applied potential.

The other two models (Figures 1C and 1D) are also possible; The CE model (Figure 1C) shares the same initial step (lithium deposition) as CC model, while the difference is that, for CE Model, lithium deposition is considered as a transient reaction that happens just at the very beginning. On the surface of deposited metallic lithium, the NH_3_ synthesis reaction cycle starts, featured by a chemical process of lithium nitridation and an electrochemical process from lithium nitride back to metallic lithium (Andersen et al., 2020). Different from the CC model, the protonation process in CE model is coupled with electron transfer, indicating an applied potential dependence. In the EC model (Figure 1D), nitrogen splitting is regarded as an electrochemical process, which is identical with the cathode reaction of a Li-N_2_ battery during discharging (Ma et al., 2017; Zhang et al., 2019). EC cycle is closed by a chemical reaction between lithium nitride and protic additive, accompanied by Li^+^ release into the solution. The above four models were proposed in previous studies based on density functional theory (DFT) calculations and indirect experimental evidences. Their validity requires further confirmation by experiments

### What are the competing reactions and how they affect the performance of NRR?

So far, HER and excessive lithium deposition are regarded as two dominating unfavorable reactions in Li-THF system (Andersen et al., 2020). In addition, lithium is known to reduce electrolytes (Li et al., 2017; Wang et al., 2020; Wu et al., 2020). Such parasitic reactions, especially the passivation of lithium by THF, could also be a significant competing reaction. Although some researchers have proposed electrolyte decomposition at extremely reducing condition (Cherepanov et al., 2021; Schwalbe et al., 2020), the reaction was not formally considered as competing reaction (Andersen et al., 2020; Lazouski et al., 2019). And its impact on performance was not well understood. Quantifying the parasitic reaction between lithium and organic solvents (THF as the most commonly used one) may provide insight into its profound influence on the nitrogen reduction process (which will be discussed in this paper).

**Figure 2:**
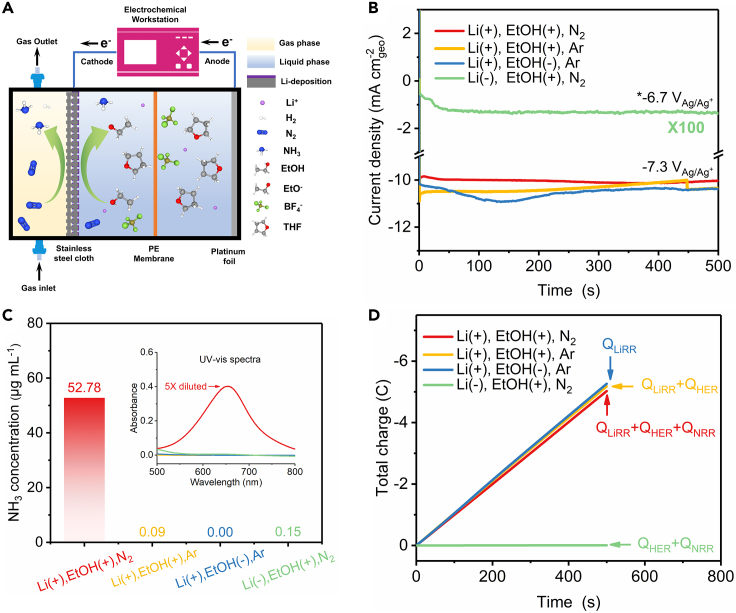
Identification of lithium-mediated NRR mechanism (A) Schematic of gas diffusion electrolytic cell with three chambers. (B) Current density profiles during chronoamperometric measurement at different conditions. ∗-6.7 V_Ag/Ag_^+^ is the most negative potential that could be provided by electrochemical working station due to dramatically increased solution resistance (Figure S5). (C) Corresponding ammonia concentration of cathode electrolyte quantified by UV-vis spectra (inset) after chronoamperometric measurement of Figure 2B. (D) Charge passed for different electrochemical process. The positive sign (+) indicates the presence of substance in electrolyte while negative sign (−) indicates the absence of substance in electrolyte.

Here, we address the above questions by a systematic investigation on the NRR mechanism in Li-THF system using a home-made gas diffusion electrolytic cell (Figure 2A). We report that in Li-THF system, lithium deposition is the predominating (>99%) electrochemical process, whereas the following nitrogen activation and protonation is basically chemical in nature. In addition, we show that the reaction between lithium deposits and THF is another important competing reaction. Only freshly deposited lithium exhibits effective reactivity to dinitrogen splitting. Once the freshly deposited lithium is passivated by THF, it gradually loses its reactivity to reduce nitrogen. Protecting “fresh” metallic lithium is therefore key to improving N_2_ reduction performance; such “fresh lithium” strategy also rationalizes how current density affects performance. We report a high ammonia yield of 0.410 ± 0.038 μg s^−1^ cm^−2^ geo and an outstanding Faradaic efficiency of 39.5 ± 1.7% at current density of 20 mA cm^−2^ geo and 10 mA cm^−2^ geo respectively, reaching top-level class in the field of electrochemical NRR at ambient condition (Tables S1 and S2).

## Results and discussion

### Identification of lithium-mediated NRR mechanism

We demonstrate that lithium-mediated NRR began with electrochemical deposition of lithium, followed by two chemical processes of dinitrogen splitting and protonation to ammonia by electrochemical measurements, which were performed with a home-made gas diffusion electrolytic cell. Stainless-steel cloth (SSC) and Pt foil were used as cathode and anode, respectively (Figure 2A and S1). Ag/Ag^+^ reference electrode was equipped to control the electrode potential accurately. The measured potential against Ag/Ag^+^ was calibrated to apparent potential against Li/Li^+^, as described in Potential calibration in Methods details. Inside the electrolytic cell, there were three chambers, denoted as gas chamber, cathode chamber, and anode chamber. Both cathode and anode chambers were filled with electrolyte and were separated by a polyethylene (PE) membrane, which retarded diffusion of ammonia from cathode to anode. A delicate gas-liquid interphase, maintained by a pressure difference between the gas chamber and the cathode chamber, was formed right on the SSC to greatly improve the mass transfer of nitrogen. The electrolytic cell was integrated with a gas purification system, an acid trap and an electrochemical workstation to form a complete device, as shown in Figure S2. We started the experiments by conducting electrolysis at a fixed potential of −7.3 V_Ag/Ag+_ (−3.56 V_Li/Li_^+^) in N_2_ saturated THF solution containing 1 M LiBF_4_ and 0.11 M EtOH (Figure 2B), which was used as the standard working condition for electrochemical ammonia synthesis. An ammonia concentration of 52.8 μg mL^−1^ in the cathode electrolyte after electrolysis (Figure 2C) was quantified by measuring the absorbance curve of the electrolyte stained with indophenol blue indicator (Figures S3 and S4). As illustrated by the red curve in Figure 2B, current density quickly reached steady state of ca. 10 mA cm^−2^ geo and kept stable in the following 500 s. The reduction current might be subscribed to lithium plating, nitrogen reduction or hydrogen evolution. To evaluate the current value that directly correlates with NRR, we replaced nitrogen gas with argon so that NRR was completely removed from the system. Surprisingly, the current curve (Figure 2B, yellow) almost coincided with the red curve (even slightly greater), suggesting that nitrogen isn't directly involved in the electrochemical process. Further, we blocked the HER by removing ethanol from electrolyte and no decrease of reduction current was observed (Figure 2B, blue), suggesting neither nitrogen nor ethanol directly take part in the electrochemical reaction to produce extra current. Hence, the reduction current here should be ascribed to lithium deposition, which was confirmed unequivocally by lithium-negative experiment whose electrolyte component was consistent with that of standard working condition except that LiBF_4_ was stripped out of electrolyte (Figure 2B, green). Electrolysis was performed at −6.7 V_Ag/Ag+_ (−2.96 V_Li/Li_^+^), which was the most negative potential that could be provided by electrochemical working station due to dramatically increased solution resistance resulting from absence of conductive ion, while the other three electrolytes containing LiBF_4_ had relative better conductivity and showed very similar electrochemical impedance spectroscopy (Figure S5). Without LiBF_4_, the electrolysis current sharply decreased to ca. 10 μA cm^−2^ geo, about 0.1% that of lithium-positive group (Figure 2B) and little ammonia was detected (Figure 2C). Figure 2D represents the amount of charge passing through the electrode surface under different conditions which correspond to different electrochemical reactions (or their combination). It is clear that lithium deposition takes the most part of charge passing through the electrode, rendering it the only dominating electrochemical process. By comparison, the charge to NRR and HER is negligible, indicating that these two reactions obtain the needed electron from deposited lithium via a chemical reaction. We also noted that the reduced charge was marginally greater in the absence of ethanol or N_2_ (Figure 2D, blue > yellow > red), indicating that the electro-deposition of lithium was slightly retarded in the presence of ethanol or N_2_. That may because ethanol and nitrogen can react with metallic lithium, leading to build-up of the by-products (such as ethoxide, lithium nitride, etc.) and thus retard the electro-deposition of lithium on the surface (Schwalbe et al., 2020).

It can be concluded that lithium-mediated NRR follows the mechanism of CC model (Figure 1A). The only dominating electrochemical process is lithium deposition while N_2_ activation and protonation, as well as hydrogen evolution, should be principally considered as the chemical processes in Li-THF system. While we recognize the possibility of electrochemical nitrogen splitting and protonation processes (Andersen et al., 2020; Schwalbe et al., 2020) (Figure 1), our results show that these electrochemical processes have limited or indiscernible contribution to the current compared with lithium plating.

The reaction between ethanol and lithium was regarded as a crucial competing reaction (Schwalbe et al., 2020), verified by performing cyclic voltammetry in electrolyte with different compositions (Figure S6). It was observed that the current of lithium-positive experiments was about three magnitudes higher than lithium-negative group (Figure S6, red and green), suggesting that the redox current here was associated with lithium. In positive sweep, the first oxidation peak located at −3 V vs Ag/Ag^+^ corresponded to oxidation of deposited metallic lithium. The oxidation peak was weakened in the presence of ethanol, suggesting the corrosion of metallic lithium by ethanol (Figure S6, red and pink). The products of reaction between metallic lithium and ethanol were estimated to be hydrogen and lithium ethoxide reported previously (Furukawa et al., 2012), which was consistent with our DFT calculation results (Figure S7).

### Parasitic reactions between deposited lithium and THF

**Figure 3:**
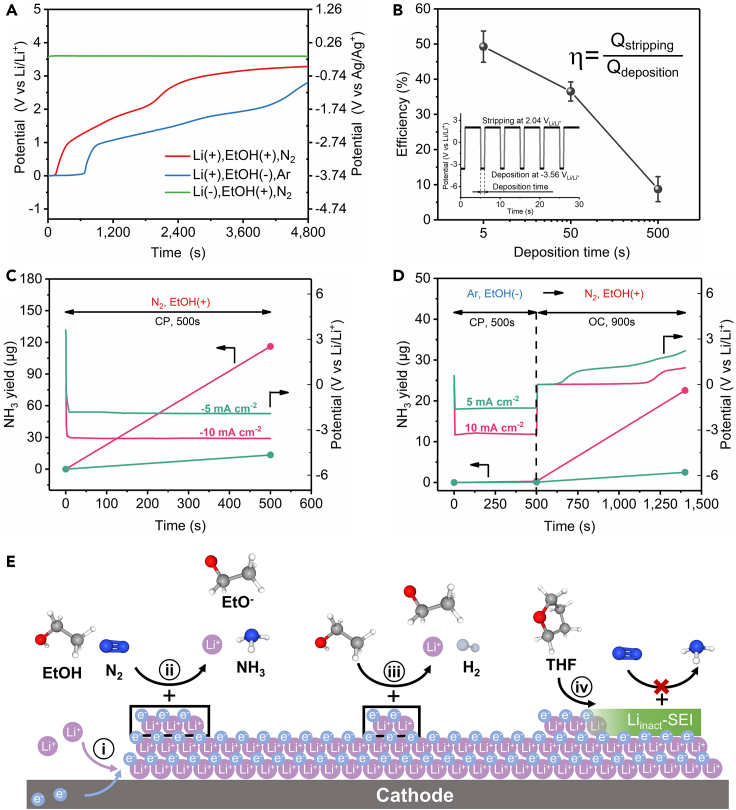
Parasitic reaction between deposited lithium and THF (A) Potential curves as a function of time at open circuit after chronoamperometric measurements in Figure 2B. The positive sign (+) indicates the presence of substance in electrolyte while negative sign (−) indicates the absence of substance in electrolyte. (B) Lithium stripping/deposition efficiency versus deposition time in THF solution only containing 1 M LiBF_4_ (free of ethanol and nitrogen) by chronocoulometric cycling between −3.56 and 2.04 V_Li/Li_^+^ (−7.3 ∼ −1.7 V _Ag/Ag_^+^). (C and D) Ammonia yield and corresponding potential profiles over time. The experiments of (C) were performed at fixed current density in N_2_ saturated electrolyte containing 0.11 M EtOH. The experiments of (D) contained two processes: electrolysis at fixed current density in Ar saturated electrolyte without ethanol followed by open circuit test in N_2_ saturated electrolyte with ethanol additive. (E) Illustration of mechanism of lithium-mediated NRR, in which four dominant phenomena seem to occur: i) electro-deposition of lithium ions, ii) chemical N_2_ splitting and NH_3_ synthesis, iii) chemical H_2_ evolution and iv) passivation of metallic Li via reaction with electrolyte.

We revealed the parasitic reactions between lithium and THF by monitoring the electrode potential at open circuit after chronoamperometric measurements in Figure 2B. For the experiments whose electrolyte containing lithium, ethanol and nitrogen, the open circuit potential originated from 0 V_Li/Li_^+^ (−3.74 V_Ag/Ag_^+^), increased gradually over time (Figure 3A). By comparison, the electrode potential of lithium-negative experiments immediately jumped to −0.16 V_Ag/Ag_^+^ due to absence of the Li/Li^+^ redox couple. For this reason, the open circuit potential can be regarded as a reliable indicator of the state of lithium on electrode. Deviation of electrode potential from 0 V_Li/Li_^+^ (−3.74 V_Ag/Ag_^+^) in open circuit potential test is an indication of corrosion of lithium deposits. Even in the electrolyte without ethanol and N_2_, the potential of lithium-deposited electrode still deviated from its thermodynamic value after a transient steady state (Figure 3A). Since the NRR and HER were excluded in the absence of ethanol and N_2_, reaction with THF is the dominant reaction to consume metallic lithium. Moreover, to avoid the possible lithium corrosion by water contaminant from ambient, we repeated the experiment in a closed electrolytic cell that was assembled and injected with electrolyte in glove box filled with Ar (Figure S8). Similar behavior of lithium corrosion in THF was observed (Figure S9). To quantify the metallic lithium consumption by THF, consecutive lithium deposition and stripping between −3.56 V_Li/Li_^+^ (−7.3 V_Ag/Ag_^+^) and 2.04 V_Li/Li_^+^ (−1.7 V_Ag/Ag_^+^) alternatively was performed in electrolyte free of ethanol and nitrogen (Figures 3B and S10). Less than 50% of the deposited Li was stripped, and the percentage dropped significantly with time, suggesting a significant occurrence of the lithium passivation reaction. During the whole process of lithium deposition/stripping, water content in electrolyte was consistently lower than 40 ppm (Figure S11), which was acceptable for lithium deposits (Koshikawa et al., 2017). In brief, our observation shows that lithium passivation by THF is a dominant reaction which shouldn't be ignored during lithium-mediated NRR study.

The interaction between lithium and THF was also corroborated by DFT calculations. We compared the reaction tendency of THF, ethanol, and nitrogen on three typical lithium facets. Focusing on the chemisorption process of reactants on lithium facets as an essential first step of the reaction between THF and metallic lithium (Figure S12), the intensity of interaction between metallic lithium and the reactants were quantified by the energy released in chemisorption process (denoted as *G*_*in*_, see Calculation method in Methods details for details). As illustrated by Figure S13, the energies released in the chemisorption process of THF, ethanol, and nitrogen are very closed on various lithium facets, validating non-negligible interaction between metallic lithium and THF.

The negative effect of THF-induced lithium reaction on the NRR performance was further evaluated by the following experiments. First, we performed electrolysis at constant current density in N_2_ saturated electrolyte containing 1 M LiBF_4_ and 0.11 M EtOH, at which ammonia was produced continuously (Figure 3C). Potential profiles were recorded and ammonia yield in electrolyte was quantified by indophenol blue method (Figure S3). Then, comparison experiments were designed to reinforce the passivation reaction which consisted of two steps: electrolysis at constant current density in an Ar saturated electrolyte without ethanol for 500 s, followed by open circuit test in N_2_ saturated electrolyte with ethanol addition for another 900 s (Figure 3D). During the first step, lithium ions deposited on electrode and underwent passivation reaction by THF. In the second step passivation reaction continued, accompanied by ammonia synthesis process. No obvious ammonia was identified in the first 500 s (Figure 3D). Nevertheless, after replacement of N_2_ with Ar and addition of ethanol, the ammonia yield of 10 mA cm^−2^ geo group increased significantly. Although no current was applied in the regime, the open circuit potential of 0 V_Li/Li_^+^ (−3.74 V_Ag/Ag_^+^) indicated that there was still residual metallic lithium on the electrode. According to the CC model (Figure 1A), N_2_ splitting process and protonation process can proceed on reactive lithium surface without applied current, which was consistent with the experimental findings. Furthermore, the CC model also helps to explain the experiment results that fewer ammonia was produced in 5 mA cm^−2^ geo group compared with that of 10 mA cm^−2^ geo group during open circuit tests. This is because fewer active lithium was left on the electrode of 5 mA cm^−2^ geo group since the open circuit potential of 5 mA cm^−2^ geo group deviated from the potential of the Li/Li^+^ redox couple much earlier than that of 10 mA cm^−2^ geo group.

Finally, the influence of parasitic reaction by THF can be demonstrated by comparing the final ammonia amount in two independent experiments (Figures 3C and 3D). It was found that ammonia yield dramatically decreased if the system went through an extra passivation period of 500 s. In the framework of the CC model (Figure 1A), the parasitic reaction consumed significant amount of active lithium which was necessary for N_2_ activation, leading to degradation of ammonia yields. On the base of above, mechanism diagram incorporating four dominant phenomena in lithium-mediated NRR is represented (Figure 3E), which clarifies the characteristic of a series of reactions (chemical or electrochemical), highlights the parasitic reaction by THF and points out the direction for optimization.

### Greater current, fresher lithium and enhanced performance

As elucidated above, keep deposited lithium fresh could be considered as a strategy for improvement of lithium-mediated NRR. The freshness of lithium deposition was measured by the speed of passivation and generation of lithium. The rate of passivation, mainly depending on intrinsic chemical properties of metallic lithium and THF, could hardly be restricted unless a more inert organic solvent than THF was employed. By comparison, the generation speed of new lithium could be easily tuned by current. In fact, lithium generation rate basically equals to reduction current since we have shown that lithium plating is the only dominating electrochemical process in Li-THF system. Hence, we speculated that both the ammonia yield rate and Faradaic efficiency to ammonia might increase with increasing electrolysis current in a certain range.

**Figure 4:**
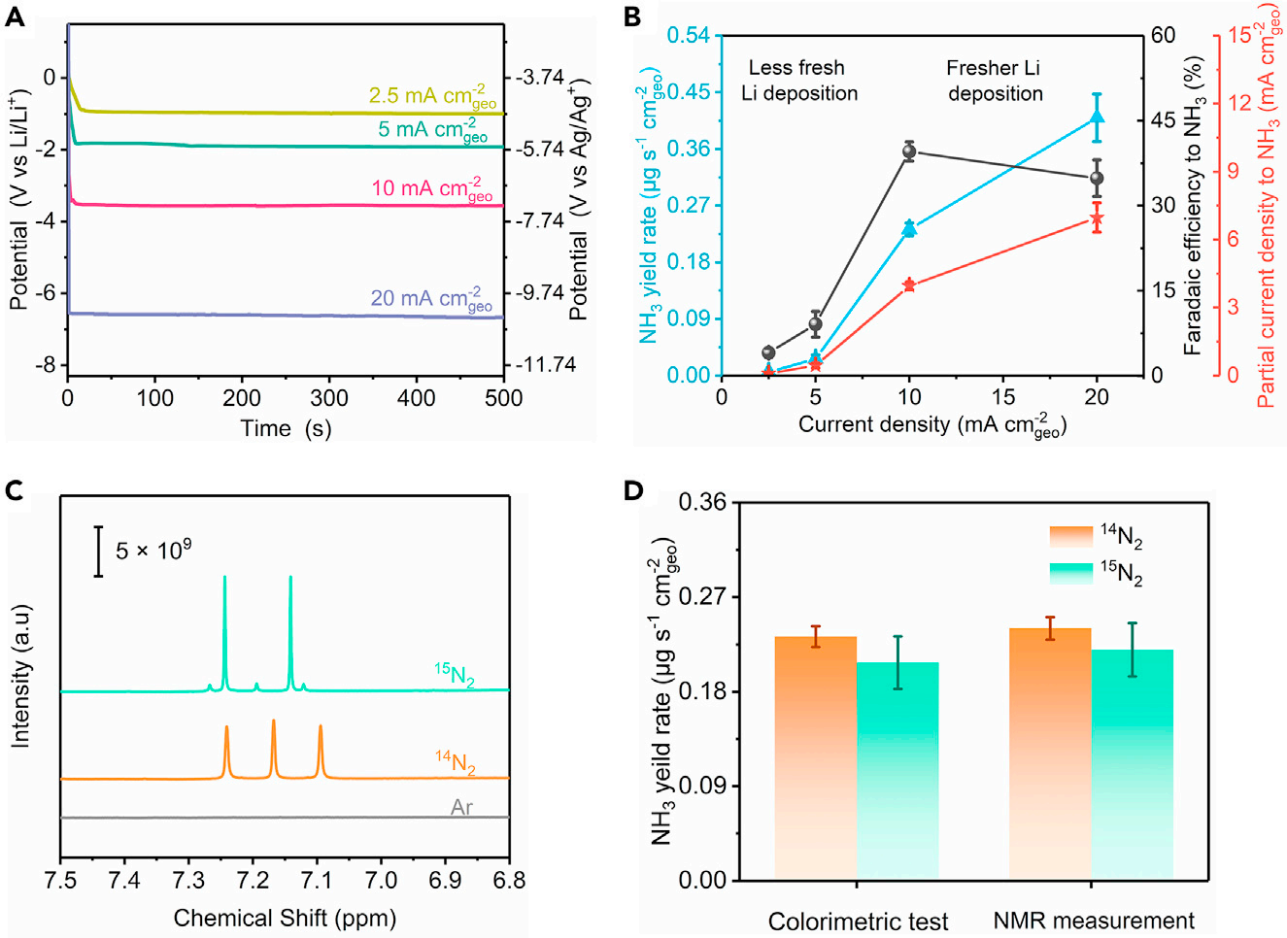
Performance of lithium-mediated nitrogen reduction reaction (A) Potential profiles during chronopotentiometric measurement at given current density. (B) Performance of lithium-mediated NRR at current density varied from 2.5 mA cm^−2^ geo to 20 mA cm^−2^ geo. (C) ^1^H NMR spectra of electrolyte after electrolysis by using ^15^N_2_, ^14^N_2_ and Ar as feed gas. (D) Comparison of ammonia yield rate quantified by both colorimetric test and NMR measurement. The error bars in Figures 4B and 4D represent standard deviation between identical electrolysis experiments (n ≥ 2).

We sought to verify the conjecture by varying electrolysis current between 2.5 mA cm^−2^ geo and 20 mA cm^−2^ geo (Figure 4A). The potential curves were stable, indicating electrolysis process quickly entered and stayed in its steady state. After electrolysis, ammonia yield rate was calculated by adding up ammonia amount in cathode chamber, anode chamber and acid trap (Cai et al., 2021), whose ammonia concentration are summarized in Figure S14. Ammonia yield rate grew monotonically with increasing current. The maximum production rate of 0.410 ± 0.038 μg s^−1^ cm^−2^ geo was reached at the current density of 20 mA cm^−2^ geo, where nitrogen mass transfer in THF is not limited by adopting a gas diffusion electrolytic cell (Lazouski et al., 2020), making it one of the best performance in NRR (Tables S1 and S2). The Faradaic efficiency to ammonia, was increased from 2.5 mA cm^−2^ geo to 10 mA cm^−2^ geo with an optimal value of 39.5 ± 1.7% achieved at 10 mA cm^−2^ geo, still outperforming most cases even in nonaqueous media. A small decrease in Faradaic efficiency was noted when current was further increased to 20 mA cm^−2^ geo, which might be attributed to the electrochemical instability, severe electrolyte decomposition and accumulation of unreacted lithium due to excessive lithium plating (Figure S15) (Andersen et al., 2020).

In short, the dependence of performance on electrolysis current was acquired, which can be explained by the freshness of lithium deposits. In electrolysis with a large plating current, lithium generation had an advantage over passivation, resulting in fresher lithium deposits and thus exposing more active lithium to nitrogen. In contrast, if electrolysis was performed at a low current, passivation prevailed over lithium generation, leading to deterioration of active lithium and thus losing its reactivity toward NRR. It is worth mentioning that, in addition to increasing current, fresher lithium can also be obtained by retarding the passivation of metallic lithium. A future strategy may involve borrowing knowledge from the battery community to create a protective layer which is impervious to electrolyte but penetrable by nitrogen and proton source via electrolyte additives and engineering the solid-electrolyte interface (Yu et al., 2020).

### Control experiments

The lithium-mediated process has been rigorously shown to fix N_2_ and synthesize ammonia (Andersen et al., 2019; Suryanto et al., 2021). Therefore, we further performed rigorous control experiments to primarily focus on ascertaining the cleanliness of our setup. The [Li(+), EtOH(+), Ar (Figure 2C, yellow)], [Li(+),EtOH(−), Ar (blue)], and [Li(−), EtOH(+), N_2_ (green)] experiments under applied potential directly quantify the extraneous ammonia & NO_x_ impurities (reduction of NO_x_ is much facile than N_2_(Choi et al., 2020b; Guo et al., 2021)) from the ethanol, electrolyte, and N_2_ gas, respectively. We also performed open circuit potential and Ar-filled control experiments and little ammonia was detected after electrolysis (Figures 3D and S16). We detect up to 0.3 μg (0.2 μg_NH3_ mL^−1^ in cathode), which are significantly smaller than the quantities (>110 μg_NH3_ at −10 mA cm^−2^ geo, Figure 3C) made in our N_2_ reduction experiments. At 20 mA cm^−2^ geo, we produce ca. 200 μg (112 μg_NH3_ mL^−1^ in cathode chamber, as illustrated in Figure S14) which significantly surpass the possible impurities from our system and the NH_3_ produced in aqueous systems (Andersen et al., 2019; Iriawan et al., 2021). We also note the quantitative similarity of our yield rate and Faradaic efficiency with a prior study (Lazouski et al., 2020) using a GDE setup in the cathodic compartment, hence showing good reproducibility of our results. We still performed quantitative ^15^N_2_ isotope experiments repeatedly (n ≥ 2), with ^15^N_2_ gas purification, to verify genuine N_2_ activation, although only at the end of electrolysis. As presented by Figure 4C, ^14^N_2_ and ^15^N_2_ caused triplet and doublet in nuclear magnetic resonance (NMR) spectra, which correspond to ^14^NH_4_^+^ and ^15^NH_4_^+^ respectively. The weak ^14^NH_4_^+^ signal in NMR spectra of ^15^N_2_ might be resulting from small amount of ^14^N_2_ in ^15^N_2_ gas (Sigma, 98 atom % ^15^N). No obvious signal was detected when Ar was applied. We calculated the ammonia yield rate by integrating of the peak areas in NMR spectra, which was linearly related to ammonia concentration (Figures S17 and S18). Results from NMR agree well with those from UV-Vis spectroscopy, regardless whether ^15^N_2_ or ^14^N_2_ was used.

### Limitations of the study

In this work, a systematic study of mechanism in lithium-mediated electrochemical nitrogen reduction was reported. The understanding of the mechanism opens up an avenue for further improvements of the system. Even though the framework of mechanism has been established in the study, the details inside are still unclear, which may require further identification of reaction intermediates on lithium deposits.

## STAR★Methods

### Key resources table

**Table:** 

REAGENT or RESOURCE	SOURCE	IDENTIFIER
**Chemicals, peptides, and recombinant proteins**		

Tetrahydrofuran	Sigma-Aldrich	CAS: 109-99-9
Lithium tetrafluoroborate	Sigma-Aldrich	CAS: 14283-07-9
silver perchlorate	Sigma-Aldrich	CAS: 7783-93-9
isotope labeled nitrogen	Sigma-Aldrich	CAS: 29817-79-6
isotope labeled ammonium chloride	Sigma-Aldrich	CAS: 39466-62-1
potassium hydroxide	Sigma-Aldrich	CAS: 1310-58-3
Ammonium chloride	Aladdin Reagent Co., Ltd	CAS: 12125-02-9
salicylic acid	Aladdin Reagent Co., Ltd	CAS: 69-72-7
sodium nitroferricyanide dihydrate	Aladdin Reagent Co., Ltd	CAS: 13755-38-9
dimethyl sulfoxide-D6	Aladdin Reagent Co., Ltd	CAS: 2206-27-1
maleic acid	Aladdin Reagent Co., Ltd	CAS: 110-16-7
Sulfuric acid	Sinopharm Chemical Reagent Co., Ltd.	CAS: 7664-93-9
trisodium citrate dihydrate	Sinopharm Chemical Reagent Co., Ltd.	CAS: 6132-04-3
Steel cloth	Golden Bug Flagship Store	CAS: 12597-68-1
Polyethylene membrane	Sunkyung Chemical Co., Ltd	CAS: 9002-88-4
Ethanol	Adamas	CAS: 64-17-5
Molecular sieves	Acros Organics	CAS: 70955-01-0
Sodium hypochlorite	General-Reagent	CAS: 7681-52-9
Ultra-high purity N_2_	Likang Gas Co., Ltd (Shanghai)	CAS: 7727-37-9
Ultra-high purity Ar	Likang Gas Co., Ltd (Shanghai)	CAS: 7440-37-1

**Software and algorithms**		

Vienna *ab initio* Simulation Program	Shanghai Jiao Tong University	https://www.vasp.at/

**Other**		

Gamry Reference 3000 Potentiostat/Galvanostat/ZRA	Gamry Instruments, Inc.	https://www.gamry.com/potentiostats/reference-3000/
831 KF Coulometer	Motrohm	https://www.metrohm.com/en-us/products-overview/karl-fischer-titration/kf-titrino-coulometers/
AVANCE NEO 700 MHz	Bruker	https://www.ucl.ac.uk/nmr/nmr-instruments/bruker-avance-neo-700
P4 Ultraviolet-visible spectrophotometer	Mapada	http://www.mapada.com.cn/productinfo/1323234.html
VEGA 3 scanning electron microscopy	Tescan	https://www.tescan.com/product/sem-for-materials-science-tescan-vega/

### Resource availability

#### Lead contact

Further information and requests for resources should be directed to and will be fulfilled by the lead contact, Junliang Zhang (junliang.zhang@sjtu.edu.cn).

#### Material availability

This study did not generate new unique reagents.

### Methods details

#### Design of electrolytic cell

To improve the mass transfer of nitrogen gas, the electrolysis experiments were performed with home-made gas diffusion electrolytic cell (Figure 2A). The cell consists of three chambers, denoted as gas chamber, cathode chamber and anode chamber. Stainless-steel cloth (SCC) was inserted between gas chamber and cathode chamber with 1 cm^2^ of area contacting the electrolyte. Effective nitrogen mass transfer was obtained by formation of delicate gas-liquid interphase on SSC. Namely, one side of SSC was in contact with nitrogen gas while the other side soaked in electrolyte. The gas-liquid interphase was maintained by pressure difference between gas chamber and cathode chamber. The pressure of cathode chamber roughly equaled to atmospheric pressure due to a small hole on the top, which connected to the atmosphere. The nitrogen in gas chamber was slightly pressurized by acid trap and a water column at the outlet of the gas chamber (Figure S2). Hence, the pressure difference between gas chamber and cathode chamber prevented electrolyte from flowing into cathode chamber. If pressure difference further increased, nitrogen flow couldn’t break through the water column at the outlet, so that it was forced to passed through the SCC instead of passing by it, which could be used to saturate the electrolyte.

#### Preparation of electrolyte

Before the preparation of electrolyte, as-purchased THF and ethanol was mixed with dried molecular sieves for at least 48 hours for further dehydration. Dried molecular sieves were obtained by heating at 350°C for 8 hours in muffle furnace filled with Ar. LiBF_4_ was dried in vacuum at 110°C for at least 8 hours, then dehydrated LiBF_4_ was quickly transferred and restored in glovebox filled with Ar. Electrolyte was prepared by dissolving LiBF_4_ in THF, followed by addition of ethanol. The operation was performed in glovebox filled with Ar. The water content of all solutions used in our experiments was quantified by via Karl-Fischer titration, which was summarized in Table S3.

#### Preparation of SSCs and PE membranes

The SCCs were cut into squares with side length of 2 cm. Then, as-prepared SSCs were cleaning by sonication in Milli-Q water for 30 minutes, followed by sonication in ethanol for another 30 minutes. The above cleaning processes were repeated for more than two times. Similarly, PE membranes were cut into rectangles with size of 3 cm × 4 cm and followed the same cleaning procedure as that of SSCs. Both SSC and PE membrane were fresh in every experiment and used only once.

#### Preparation of reference electrode

Ag/Ag^+^ reference electrode was adopted in our experiments. The Ag/Ag^+^ reference electrode was made by placing a clean silver wire into an electrolyte containing silver ion which was sealed in a glass tube. The electrolyte in the reference compartment was prepared by dissolving 0.1 M AgClO_4_ in in THF. After that, the electrolyte was injected into glass tube by syringe. Since AgClO_4_ solution easily decomposes in light and absorbs water from ambient, we reprepared the electrolyte before every experiment.

#### Gas circuit setup

The entire gas circuit setup is presented in Figure S2, which can be divided in to two parts: gas pretreatment device and an acid trap downstream of electrochemical cell. The function of gas pretreatment device is to provide THF saturated nitrogen gas free of water and N-containing impurities. And that was realized by four gas-washing bottles. The first gas-washing bottle containing 0.05 M H_2_SO_4_ was used to eliminate alkaline N-containing impurities, including ammonia. After that, the gas flow was conducted into the second gas-washing bottle filled with 0.1 M KOH to remove acidic N-containing impurities like NO_x_. Then, purified nitrogen went through THF solution mixed with dried molecular sieves, which were sealed in the third gas-washing bottle. The dried molecular sieves were used to absorb water vapor that was introduced by the first two gas-washing bottles. The dehydration process is necessary because water may cause performance degradation in lithium-medium system (Lazouski et al., 2019). Thorough water removal of feed gas was ensured by adding another dehydration bottle with the same configuration.

To accurately determined the ammonia production rate, acid trap filled with 12 mL 0.05 M H_2_SO_4_ was connected to the outlet of electrolytic cell to absorb the ammonia in tail gas. Ammonia amount in gas phase was estimated by measuring ammonia concentration in absorption solution. At the end of gas circuit, there was a measuring cylinder filled with water. The pressure of gas flow was tuned by the height of water column in measuring cylinder.

#### Electrochemical measurements

In the day before electrochemical experiment, all components, including gas tube, assembly parts of Ag/Ag^+^ reference electrode, Pt counter electrode, electrolytic cell and its seal assembly were rinsed successively by ethanol, tap water and Milli-Q water. Then, these components as well as SSC and PE membrane were transferred to oven and dried at 75°C under vacuum for overnight. After that, electrolytic cell was assembled, connected to gas circuit and tested at ambient condition. In a typical experiment, 5 standard cubic centimeters per minute (sccm) of N_2_ were bubbled through the entire setup for at least 10 minutes, removing the residual air in pipeline. Next, 1.5 mL of electrolyte was added to each chamber. Electrolyte was saturated with feed gas by means of increasing backpressure by immersing final outlet of pipeline deeper in measuring cylinder so that nitrogen flow changed its direction, flowed through the SSC and escaped through a small hole on the top of cathode chamber (See the Design of electrolytic cell for details). The saturation process lasted for 10 minutes at flow rate of 5 sccm. At the end of the preparation stage, backpressure was decreased so that nitrogen flow could no longer pass through it, but simply pass it. A delicate gas-liquid interphase was formed right on the SSC. Electrochemical measurements were performed using Gamry Reference 3000 electrochemical workstation. The electrolysis was performed at constant current/potential for 500 s, sometimes followed immediately by open circuit potential test. During the test, the rate of nitrogen flow was kept constant at 5 sccm, except for long term open circuit potential test, in which nitrogen flow rate was further decreased to ca. 1 sccm.

For chronocoulometric cycling of lithium deposition/stripping (Figures 3B and S10), a more rigorous procedure was required to minimize the potential interference of water and oxygen in air (since they are known to cause lithium corrosion). To be specific, the cell was assembled and injected with electrolyte in glovebox filled with Ar. Then, the cell was completely sealed to keep the water and oxygen out before it was transferred outside the glovebox for lithium deposition/stripping test. After electrochemical experiments, the cell was transferred back to glovebox for water quantification.

#### Potential calibration

In our experiments, Ag/0.1 M Ag^+^ reference electrode was employed as reference electrode, by which the potential of working electrode was measured/controlled. Then measured potential against Ag/0.1M Ag^+^ (abbreviated as Ag/Ag^+^) was calibrated to apparent potential against Li/1 M Li^+^ (abbreviated as Li/Li^+^). The Li/Li^+^ redox potential against that of Ag/Ag^+^ was determined by three independent methods, which fit pretty well with each other. 1) Open circuit potential test. The immediate potential measurement of electrode deposited with metal lithium was found to be −3.74 V vs Ag/Ag^+^. 2) Cyclic voltammetry. As presented in Figure S19, the onset potential of lithium plating is estimated to be −3.74 V vs Ag/Ag^+^ in cyclic voltammetry curve with IR-correction where R was estimated by electrochemical impedance spectroscopy (Wei et al., 2019) (EIS). 3) Calculation from literature. According to value provided by literature (Gritzner, 2010), the potential of metallic lithium in 1 M Li^+^ in THF was calculated to be −3.75 V vs Ag/0.1 M Ag^+^ reference electrode. Thus, the Li/Li^+^ redox potential was determined to be −3.74 V vs Ag/0.1 M Ag^+^.

#### ^15^N_2_ isotope labeling experiments

The isotope experiments were conducted following the similar procedure. Firstly, 5 sccm of Ar were bubbled through the entire setup for at least 10 minutes, followed by addition of 1.5 mL of electrolyte to each chamber. Secondly, Ar was conducted to flow through the electrolyte at rate of 5 sccm for 10 minutes, replacing all impurity gas molecules with argon. Then, Ar was replaced by ^15^N_2_. The saturation process continued for another 10 minutes. After the electrolyte was fully saturated by ^15^N_2_, the backpressure was decreased and ^15^N_2_ began to flow pass by the electrode. The flow rate was kept constant at 5 sccm during electrochemical test.

#### Quantification of ammonia

The concentration of ammonia in electrolyte was quantified by indophenol blue method and nuclear magnetic resonance (NMR) measurements. We calculated ammonia yield rate by considering all the ammonia from electrolyte and tail gas adsorber. Since the ammonia amount in our electrolyte was 1–2 orders of magnitude higher than that of literature, test protocols were modified to meet the requirement.

For indophenol blue method, 0.1 mL of electrolyte was mixed with 1 mL of 1 M NaOH solution containing 5 wt.% salicylic acid and 5 wt.% sodium citrate, followed by successive addition of 0.5 mL of 0.05 M NaClO solution, 0.1 mL of aqueous solution of 1 wt.% sodium nitroferricyanide and 0.9 mL Milli-Q water. The obtained solution was transferred to centrifuge tube and centrifuged at 11,000 revolutions per minute (r.p.m.) for 15 min to precipitate insoluble impurities, which may influence UV-vis absorbance. At 1 hour from the time indophenol indicator was added, absorption spectrum (background correction included) ranged from 800 nm to 450 nm was obtained using UV-vis spectrometer (Mapada, P4). Ammonia concentration was acquired by substituting absorbance difference between 655 nm and 800 nm (A655-A800) into calibration curve. The calibration curve was updated for every measurement. To evaluate the possible influence of electrolyte decomposition products on the indophenol reaction, we rechecked our results by adopting the internally calibrated indophenol blue method recommended by reference (Suryanto et al., 2021), which fits well with our method (Figure S4). It should be noted that the scheme is suitable for electrolyte with ammonia amount in range of 0–25 μg mL^−1^. For sample with higher ammonia concentration, 20 μL of electrolyte was diluted 5 times with blank electrolyte before the addition of indophenol indicator. Besides, the quantification of ammonia in solution of acid trap followed the classical protocol for aqueous electrolytes. 1 mL solution sample was added to 1mL of 1 M NaOH solution containing 5 wt.% salicylic acid and 5 wt.% sodium citrate. Then, 0.5 mL of 0.05 M NaClO solution and 0.1 mL of 1 wt.% sodium nitroferricyanide solution were added in sequence.

In NMR measurements, maleic acid and DMSO-d_6_ were employed as internal standard and deuterium reagent, respectively. In a typical procedure, 250 μL of electrolyte sample was mixed with 25 μL of 0.1M H_2_SO_4_ aqueous solution, 25 μL of 3 mM maleic acid solution (10 vol.% H_2_O +90% vol.% DMSO-d_6_) and 450 μL of DMSO-d_6_, with total volume of 750 μL. Then, the as-prepared solution was sealed in nuclear magnetic tube and tested by Bruker AVANCE NEO spectrometer operating at a ^1^H frequency of 700.23 MHz. Spectra was required by zg30 program with 128 scans. To accommodate the lower ammonia concentration in solution of acid trap (0.05 M H_2_SO_4_ aqueous solution) and minimize the influence of water (excessive water might cause peak distortion in NMR spectra), the protocol of NMR measurements for solution samples in acid trap was different from that used for THF-based electrolyte. 100 μL of absorbent solution was mixed with 625 μL of DMSO-d_6_, followed by addition of 25 μL of 3 mM maleic acid solution (10 vol.% H_2_O +90% vol.% DMSO-d_6_). Zg30 program with 512 scans was employed.

##### Morphology characterizations

The morphology of lithium deposits was characterized by TESCAN VEGA 3 Scanning Electron Microscopy (SEM), with an electron emission source of LaB_6_ operated at 20 kV (Figure S20). To avoid potential damage to morphology caused by water and oxygen, electrolytic cell was immediately transferred to glove box filled with Ar after electrolysis. Then we took the electrode out of cell, cut it into suitable size for measurement. On the day of measurement, the electrode pieces were stuck on detachable sample stages of SEM with the side that touched electrolyte up. Afterward, the sample stages were preserved in sealed plastic bag. Until this stage, all operations for electrodes were finished in glove box. Finally, by the side of SEM, we took the sample stages out of plastic bag and transferred them into sample chamber of SEM immediately. Even though the samples were shortly expose to air during transfer process, we believe the effect was limited.

##### Calculation method

All calculations were performed by DFT on Vienna *ab initio* Simulation Program (VASP) (Kresse and Furthmuller, 1996a, 1996b; Kresse and Hafner, 1993, 1994). The PBE functional with projector augmented wave pseudo-potential was applied on all models (Blochl, 1994; Kresse and Joubert, 1999). A Gaussian smearing technique was used with a smearing parameter of *k*_*B*_*T* = 0.1 eV for the fractional occupation of the one-electron energy levels to accelerate SCF convergence and all calculated energies were extra-polated to *k*_*B*_*T* = 0 eV.

The interaction between lithium surface and THF, ethanol and nitrogen were calculated on typical lithium facets including (100), (110) and (111) facets. All slab models were consisted of 4 layers and all slabs were fixed except top slab. (110) facet model was sampled by a Monkhorst-Pack k-point net of 7 × 5 × 1 and 5 × 5 × 1 was adopted on (100) and (111) facet models. A vacuum slab of 20 Å and cutoff energy of 500 eV were employed. Molecule calculations were sampled by Gamma point.

The free energy was calculated as followed (Norskov et al., 2004):

G=E+ZPE−TS

where *E* is the DFT energy, ZPE is the zero-point energy which was calculated by *∑(hv*_*i*_*/2)* (*h* is the Planck constant and *v*_*i*_ is the vibrational frequency), *T* is the temperature (298.15 K), *S* is the entropy of the structure determined by vibrational frequency (Norskov et al., 2004).

Because the interaction between lithium and electrolyte is related to the complicated formation process of Solid-Electrolyte Interface (SEI), the decomposition processes of adsorbates were not considered in calculation. The intensity of interaction was quantified by:

$$G_{in}=\;G*-G_{slab}-G_{molecule}$$

where *G*_*∗*_ is the free energy of the chemisorption structure, *G*_*slab*_ is the free energy of clean lithium slab model and *G*_*molecule*_ is the free energy of THF, ethanol or nitrogen. It is worth mentioning that the energies of *ZPE* and *TS* are ignored in the calculation of *G*_*slab*_. The larger *G*_*in*_ is, the interaction is stronger.

## Data Availability

All data is available in the main text or the supplemental information. Any additional information is available from the lead contact on request.

## Supporting citations

The following references appear in the Supplemental Information: Lazouski et al., 2019; Akira et al., 1994; Andersen et al., 2019; Gao et al., 2020; Schwalbe et al., 2020; Andersen et al., 2020; Lee et al., 2018; Tsuneto et al., 1993; Lazouski et al., 2020; Zhou et al., 2017; Kim et al., 2016; Pappenfus et al., 2009; Suryanto et al., 2018, 2021; Shi et al., 2017; Lv et al., 2018; Li et al., 2019; Ba et al., 2020; Wang et al., 2018a, 2018b; Tong et al., 2020; Xu et al., 2020; Yang et al., 2018; Lan and Tao, 2013; Tao et al., 2019; Luo et al., 2019; Wu et al., 2019.
