# Supplementary material for: Lithium-mediated electrochemical nitrogen reduction: Mechanistic insights to enhance performance
Source: iScience. 2021 Sep 9;24(10):103105. doi: 10.1016/j.isci.2021.103105 (PMC8482511; doi:10.1016/j.isci.2021.103105)
Supplement: Document S1. Figures S1–S20 and Tables S1–S3 [file mmc1.pdf]

iScience, Volume *24*

Supplemental information

Lithium-mediated electrochemical nitrogen reduction: Mechanistic insights to enhance performance

Xiyang Cai, Cehuang Fu, Haldrian Iriawan, Fan Yang, Aiming Wu, Liuxuan Luo, Shuiyun Shen, Guanghua Wei, Yang Shao-Horn, and Junliang Zhang

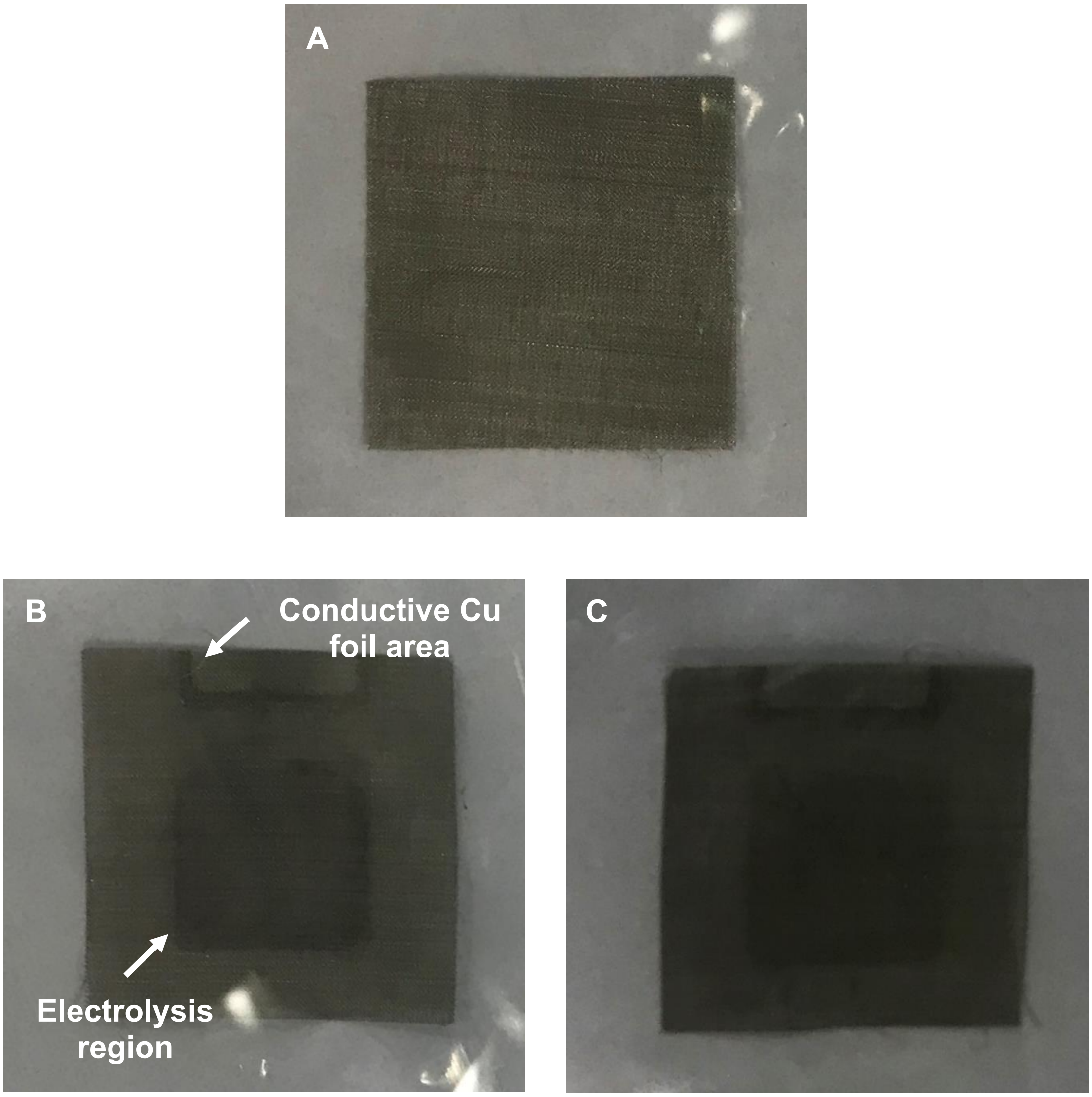

Figure S1. Related to Figure 2. Photographs of stainless-steel cloths (SSCs) used as working electrodes in our experiments. The SSCs were preserved in sealed plastic bags to keep them from contaminants. (A) New SSC before electrolysis. (B) The electrolyte side of SSC after electrolysis. (C) The gas side of SSC after electrolysis.

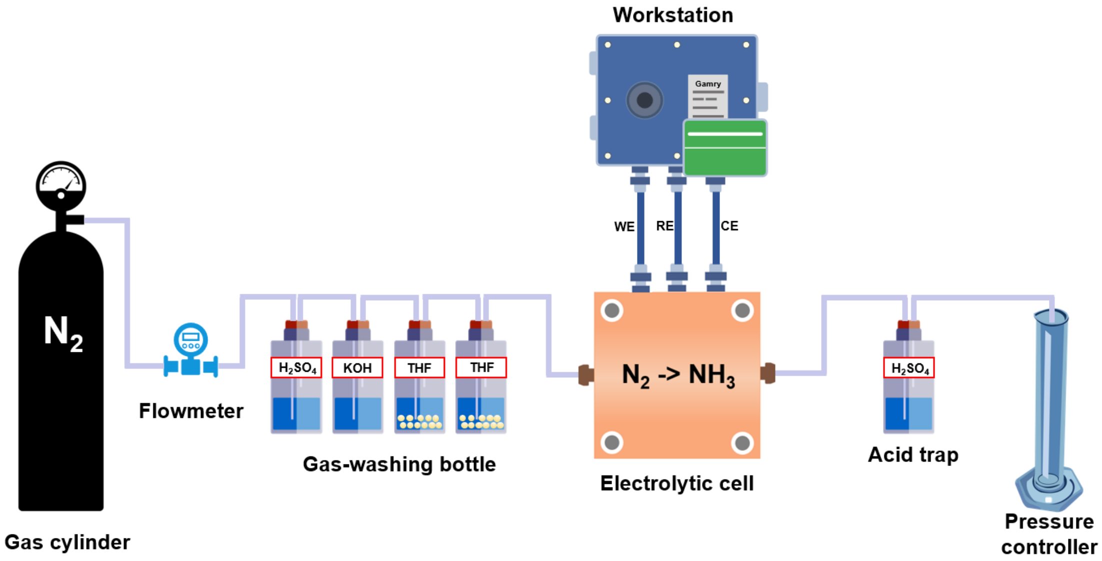

Figure S2. Related to Figure 2. Schematic of gas circuit setup in experiments.

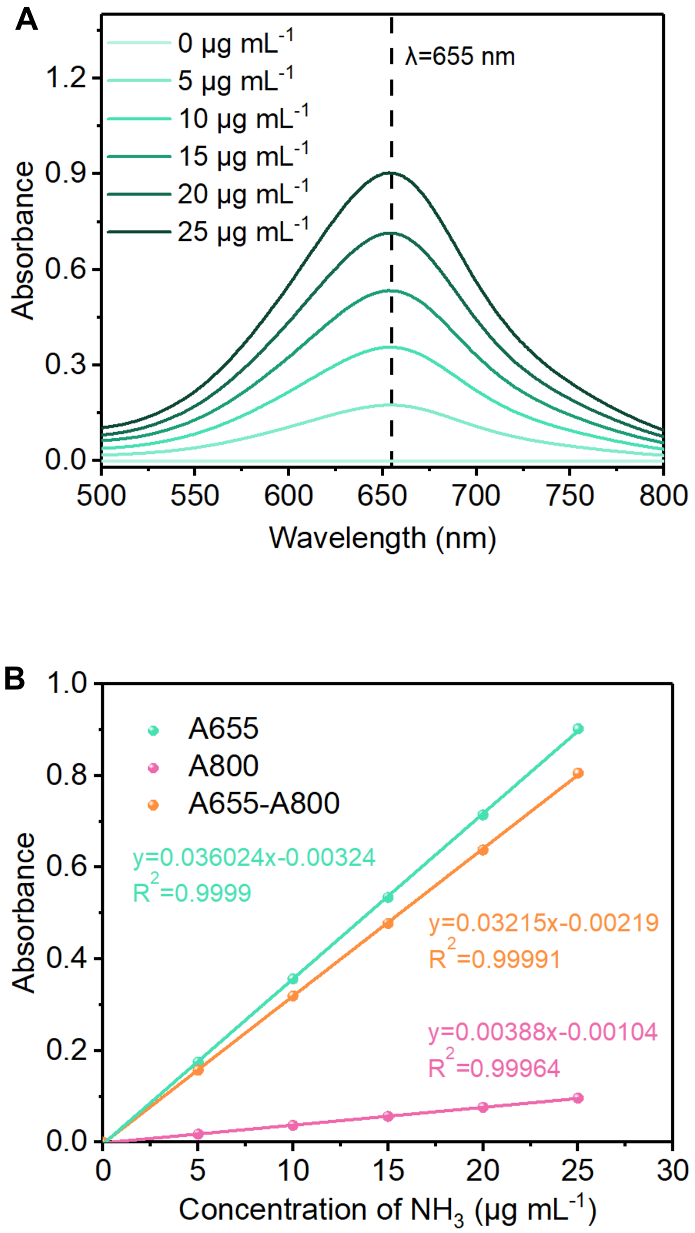

Figure S3. Related to Figure 2. Quantification of ammonia by indophenol blue method. (A) UV-vis spectra of indophenol indicator-stained electrolyte with various known ammonia concentration. It should be noted that background correction was included by subtracting absorbance of blank solution. (B) Corresponding calibration curves made by plotting absorbance values at 655 nm, 800 nm and the absorbance difference between 655 nm and 800 nm versus ammonia concentration. They all show good linear relationship with ammonia concentration. Since unknown substance produced during electrolysis will cause overall rise of absorbance curve, absorbance difference between 655 nm and 800 nm was employed as effective signal and substituted into corresponding calibration curve(Lazouski et al., 2019).

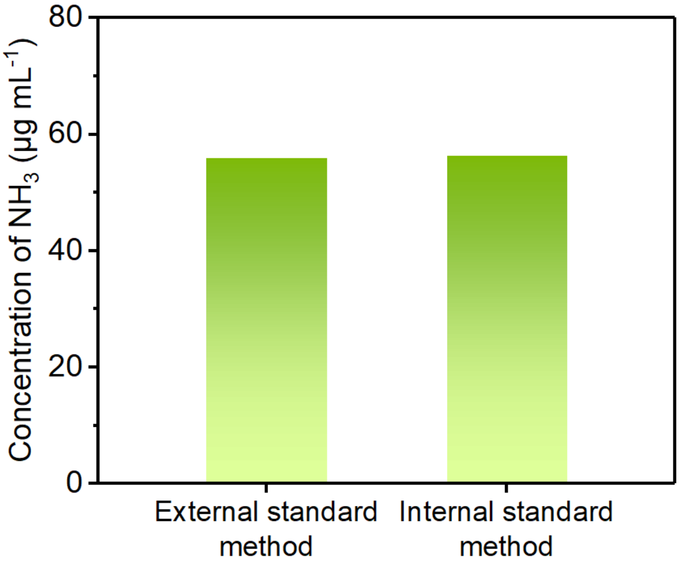

Figure S4. Related to Figure 2. Comparison of ammonia quantification results of indophenol blue method by using external calibration (used in our experiments) and internal calibration (suggested by Suryanto et al., 2021).

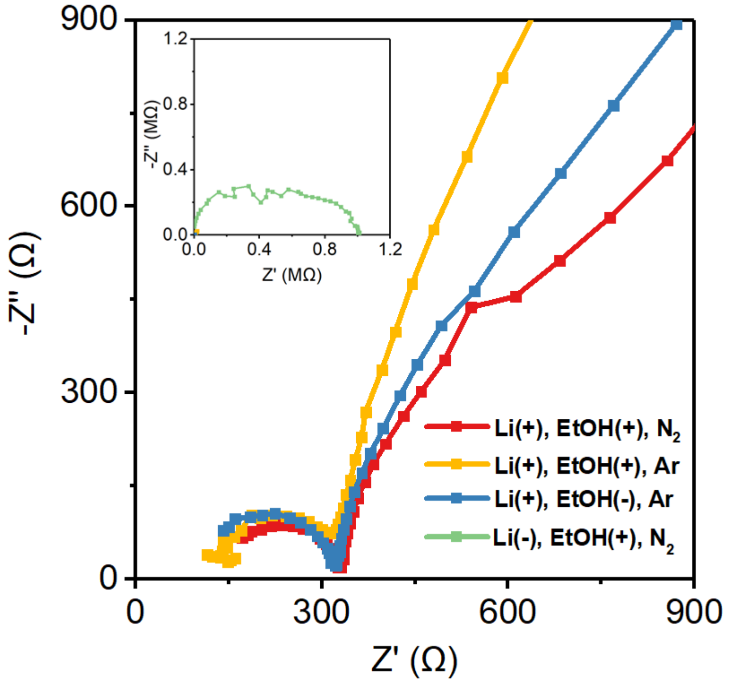

Figure S5. Related to Figure 2. Electrochemical impedance spectroscopy (EIS) at open circuit voltage at given electrolyte composition. The EIS was conducted before any electrochemical test.

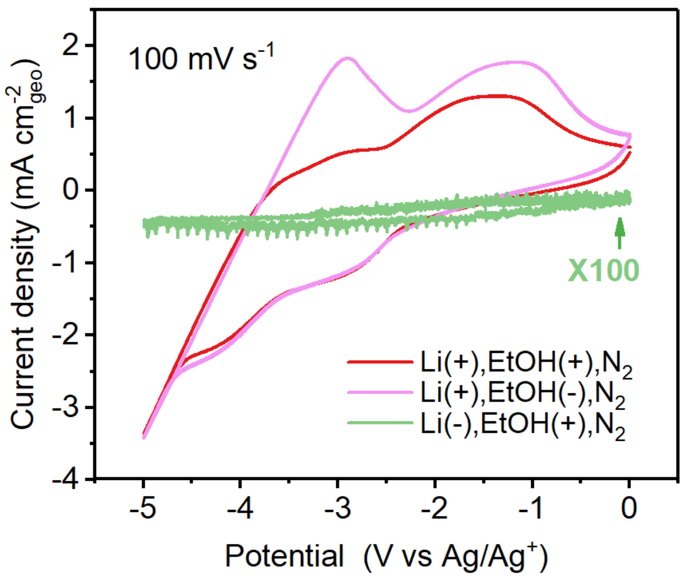

Figure S6. Related to Figure 2. Comparison of cyclic voltammetry test of SSC electrode in electrolyte with different compositions. The positive sign (+) indicates the presence of substance in electrolyte while negative sign (-) indicates the absence of substance in electrolyte. Reported potentials were not corrected for ohmic (IR_u) losses.

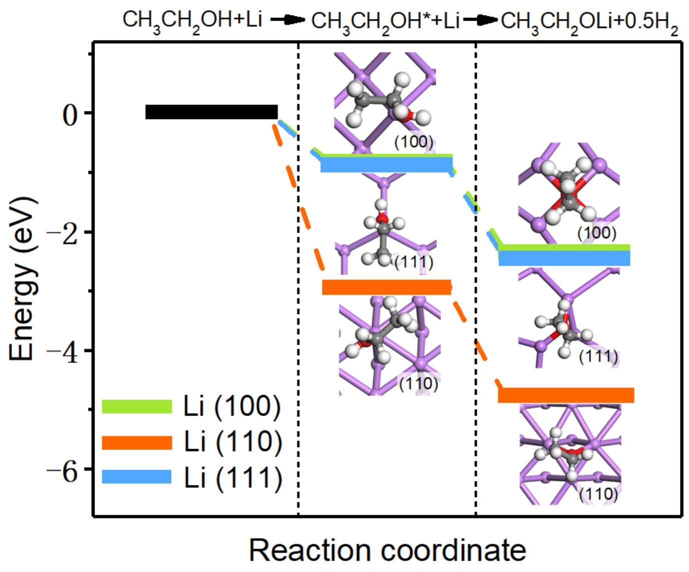

Figure S7. Related to Figure 2. The energy diagram of the hydrogen evolution reaction between lithium and ethanol. Purple, white, gray and red balls represent lithium, hydrogen, carbon and oxygen atoms.

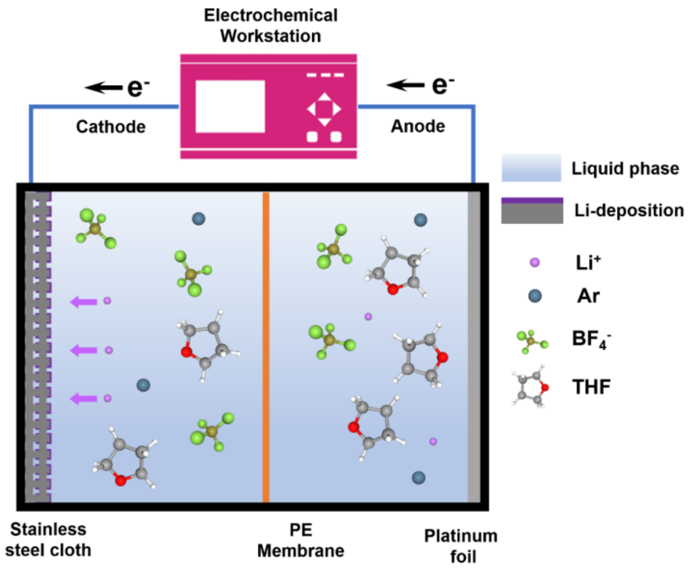

Figure S8. Related to Figure 3. Schematic of closed electrolytic cell with two chambers. The cell was assembled and injected with electrolyte in glovebox filled with Ar. Then, the cell was well sealed and moved out of glovebox where electrochemical measurements was conducted.

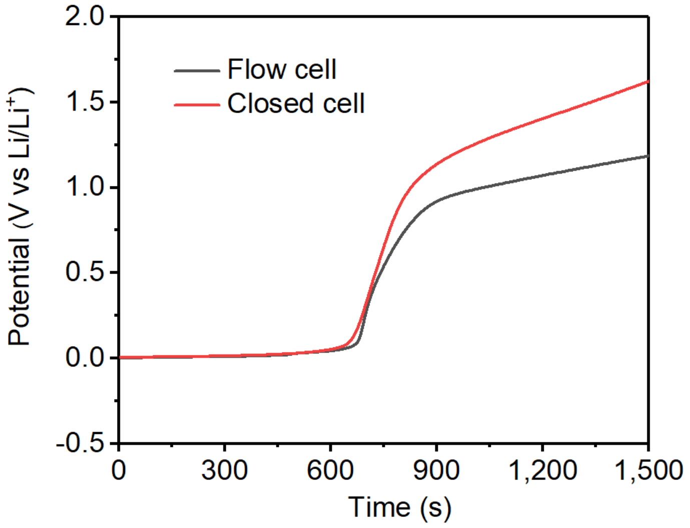

Figure S9. Related to Figure 3. Comparison of open circuit potential curves after electrolysis in flow cell (Figure 2A) or closed cell (Figure S8), respectively. Open circuit potential tests were performed immediately after electrolysis at -3.56 V_{Li/Li^+} (-7.3 V_{Ag/Ag^+}) for 500 s in Ar saturated electrolyte only containing 1 M $LiBF_4$.

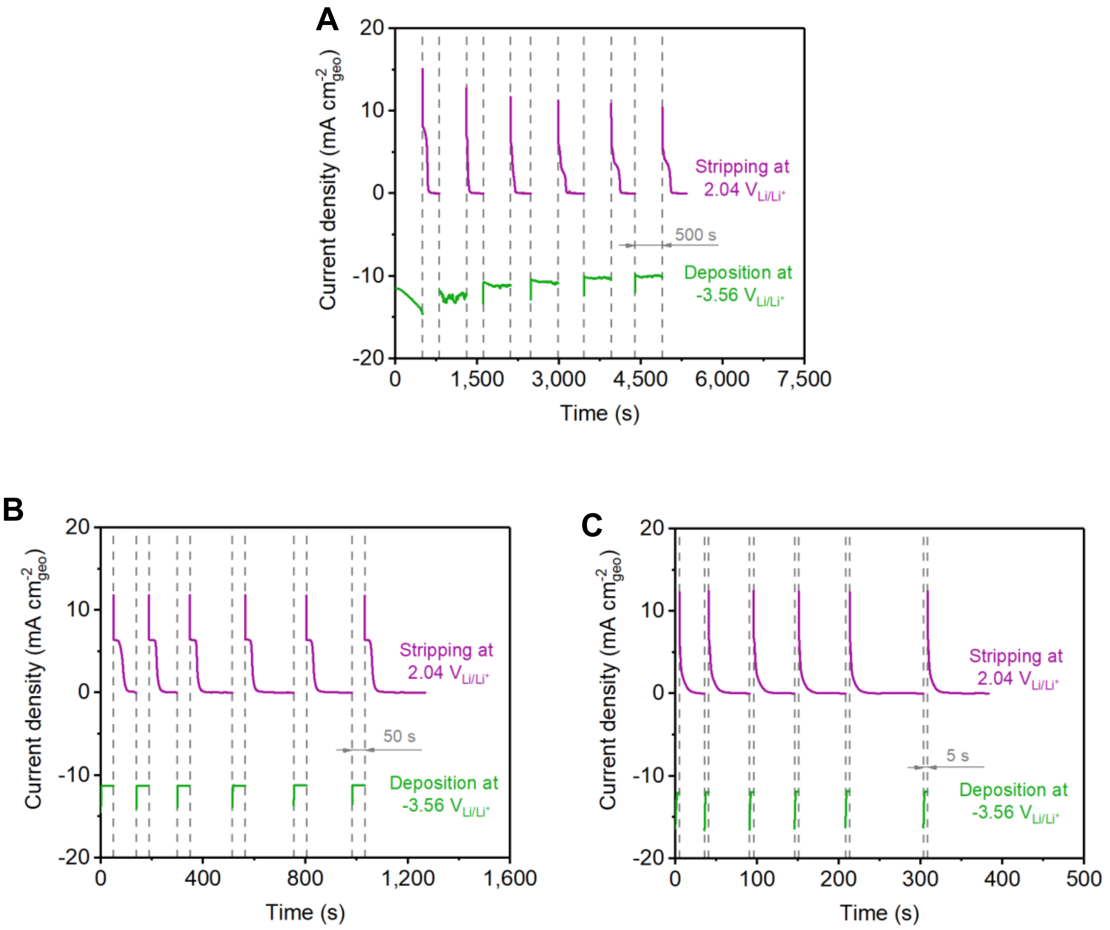

Figure S10. Related to Figure 3. Lithium deposition and stripping between -3.56 V_{Li/Li^+} (-7.3 V_{Ag/Ag^+}) and 2.04 V_{Li/Li^+} (-1.7 V_{Ag/Ag^+}) alternatively in electrolyte only containing 1 M $LiBF_4$ (free of ethanol and nitrogen). Deposition time was set as (A) 500 s, (B) 50 s and (C) 5 s The stripping potential is 2.04 V higher than potential of metal lithium to make sure all active lithium was wiped. The terminal point of lithium stripping is when current changed from positive to negative. Experiments were carried out in closed electrolytic cell (Figure S8).

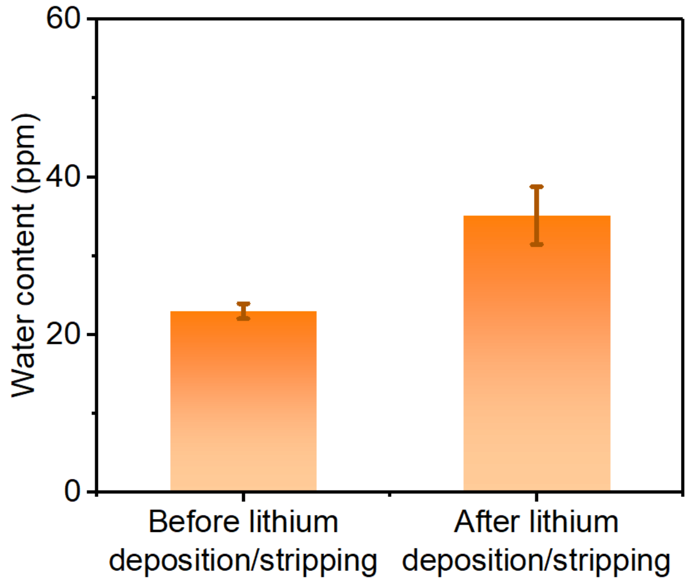

Figure S11. Related to Figure 3. Water content in electrolyte before and after lithium deposition/stripping cycles.

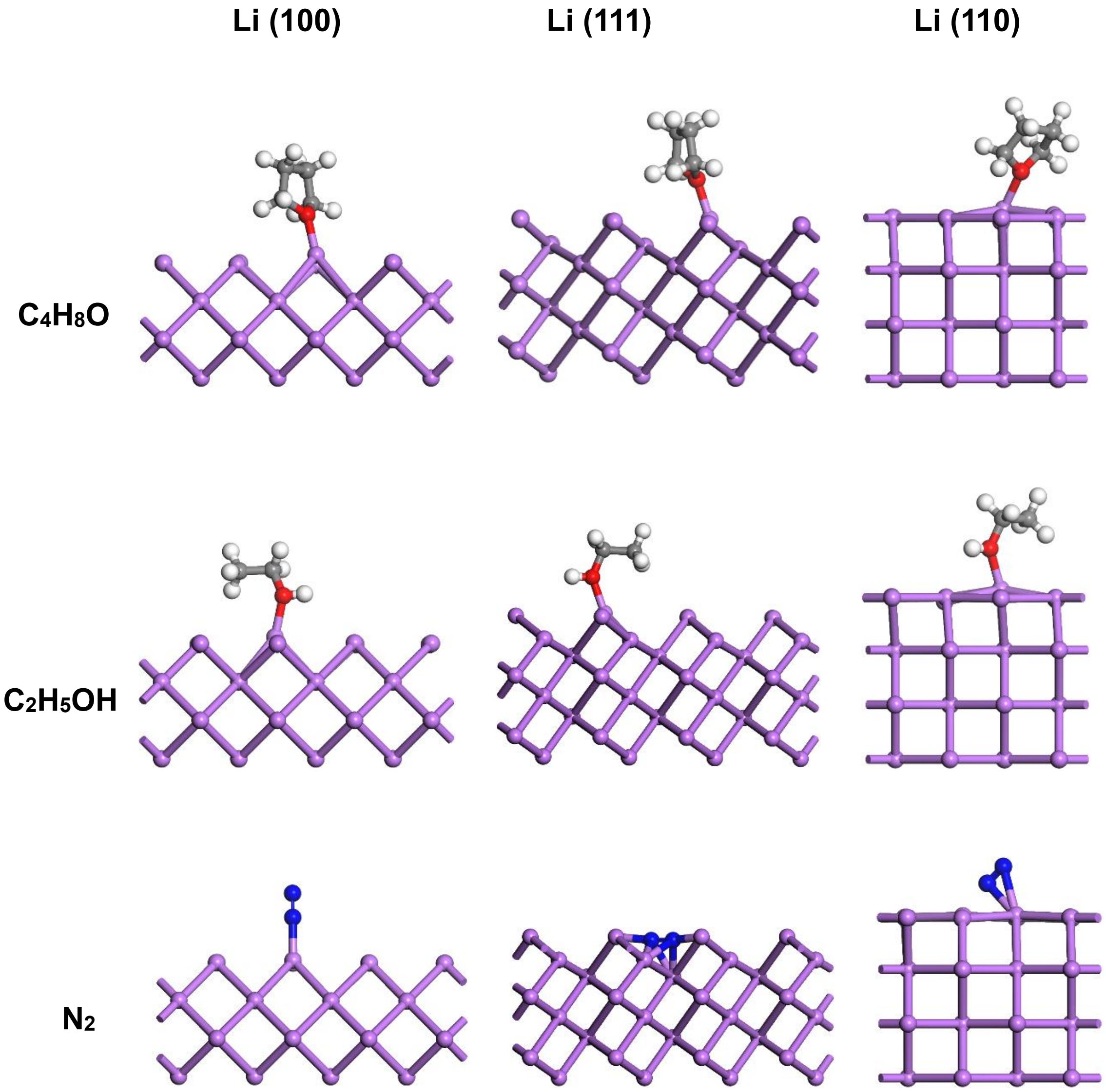

Figure S12. Related to Figure 3. Illustration of typical lithium facets interacted with THF, ethanol and nitrogen. Purple, white, gray and red balls represent lithium, hydrogen, carbon and oxygen atoms.

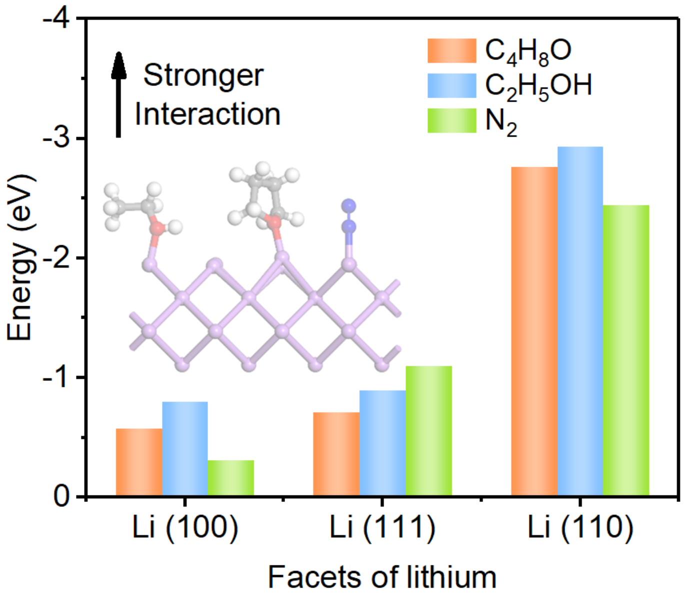

Figure S13. Related to Figure 3. The energy released in chemisorption process (G_{in}) on lithium (100), (111) and (110) facet estimated by DFT calculation.

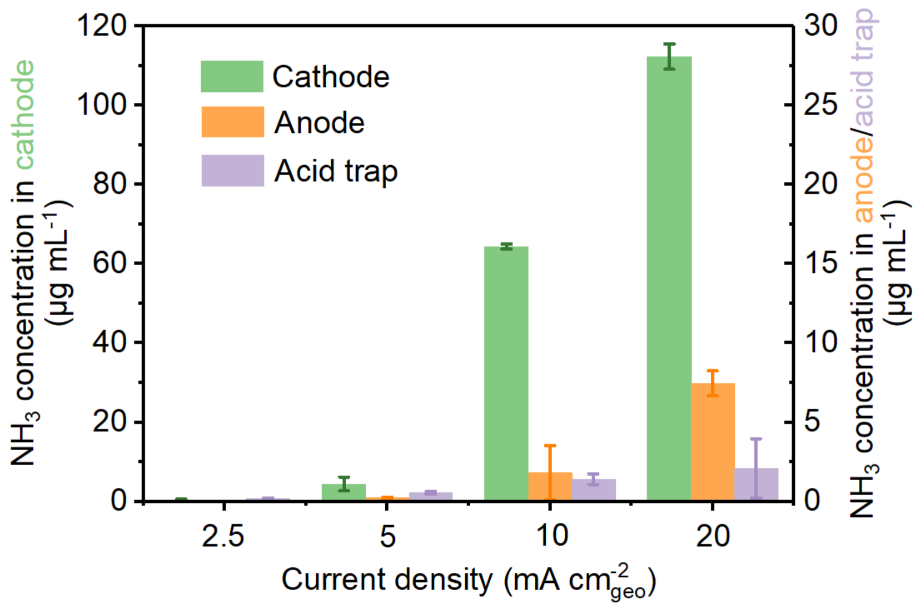

Figure S14. Related to Figure 4. The concentration of produced ammonia in cathode chamber, anode chamber and acid trap after electrolysis at given current density in N_2 saturated THF solution containing 1 M $LiBF_4$ and 0.11 M EtOH (Figure 4A). Even though PE membrane was employed as a separator between cathode chamber and anode chamber, produced ammonia still diffused from cathode to anode.

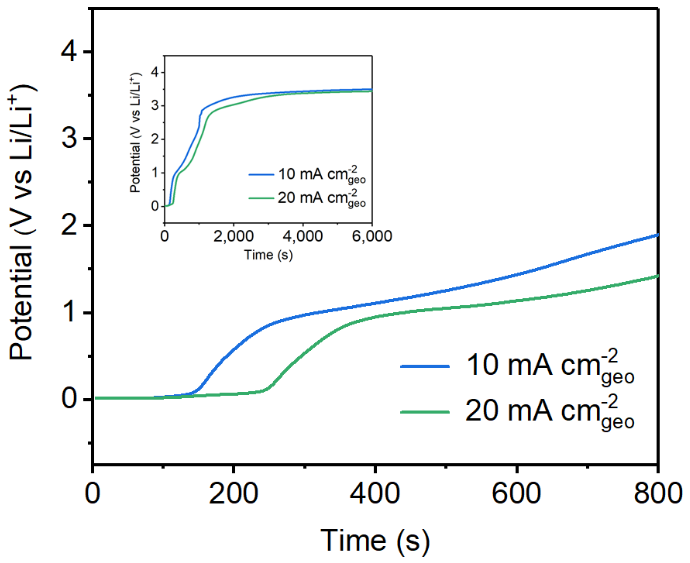

Figure S15. Related to Figure 4. Comparison of open circuit potential curves after electrolysis at given current density for 500 s.

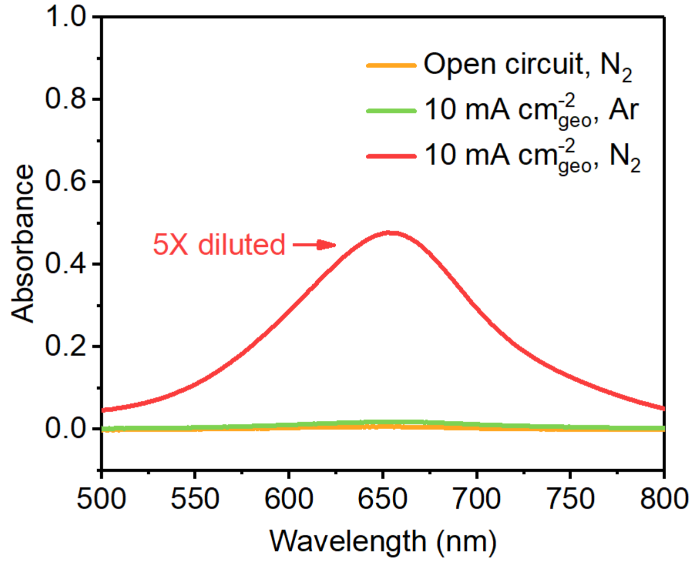

Figure S16. Related to Figure 4. Comparison of UV-vis absorbance curves of electrolyte stained with indophenol indicator after experiments at given conditions. Background correction was included by subtracting absorbance of blank solution. No obvious ammonia was found in control experiments. In comparison, distinct peak was detected when electrolysis was performed at normal condition, even though the electrolyte was further diluted for 5 times before colorimetric test.

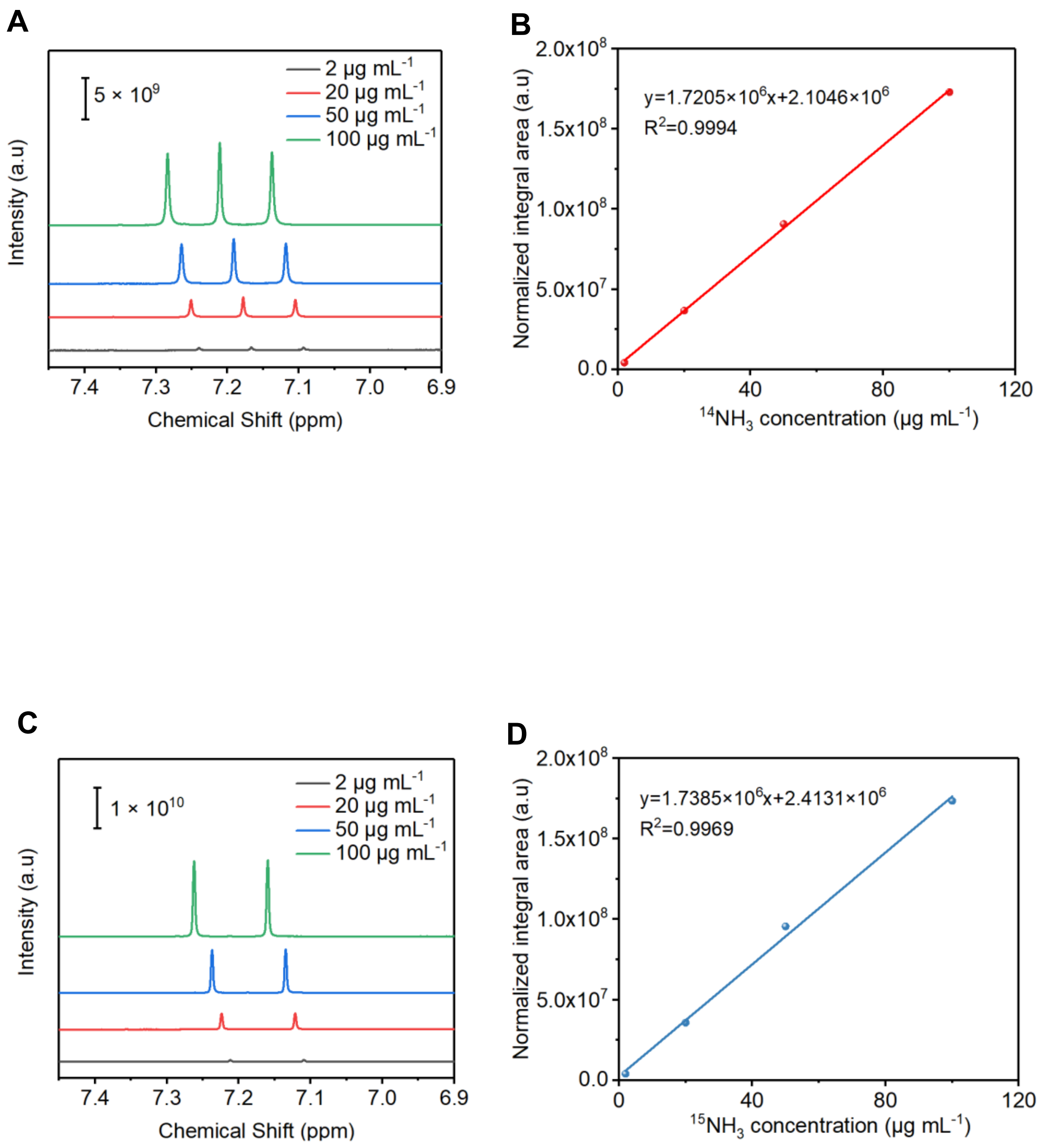

Figure S17. Related to Figure 4. Quantification of ammonia by nuclear magnetic resonance (NMR). (A,C) NMR spectra of samples with varied concentration of $^{15}NH_3$ and $^{14}NH_3$. (B,D) Calibration curve made by plotting peak area versus ammonia concentration.

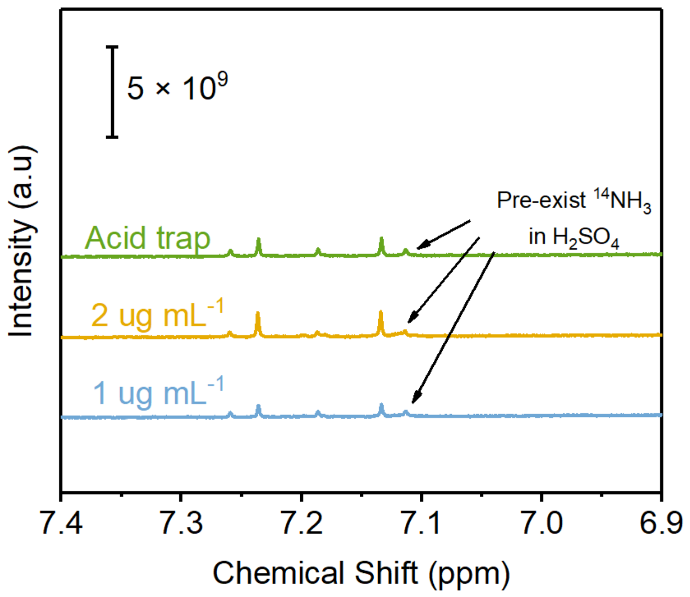

Figure S18. Related to Figure 4. NMR spectra of 0.05 M H_2SO_4 aqueous solution in acid trap after $^{15}N_2$ isotopic experiment and 0.05 M H_2SO_4 aqueous solution with known concentration of $^{15}NH_3$. To accommodate the lower ammonia concentration in acid trap and minimize the influence of water (excessive water might cause peak distortion in our experiments), the protocol of NMR measurements for samples here was different from that used for THF-based electrolyte (see **Quantification of ammonia** in **METHODS DETAILS** for details). Triple peaks of the same intensity in the figure were ascribed to pre-exist $^{14}NH_3$ in H_2SO_4, which was not considered in ammonia quantification.

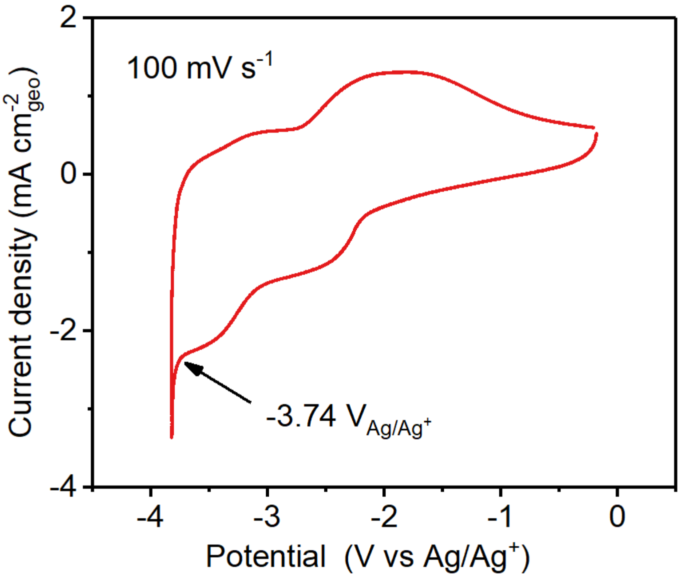

Figure S19. Related to STAR Methods. Cyclic voltammetry curve with iR-correction at a scan rate of 100 mV s^{-1}. The experiment was conducted in N_2 saturated electrolyte containing 1 M $LiBF_4$ and 0.11 M EtOH.

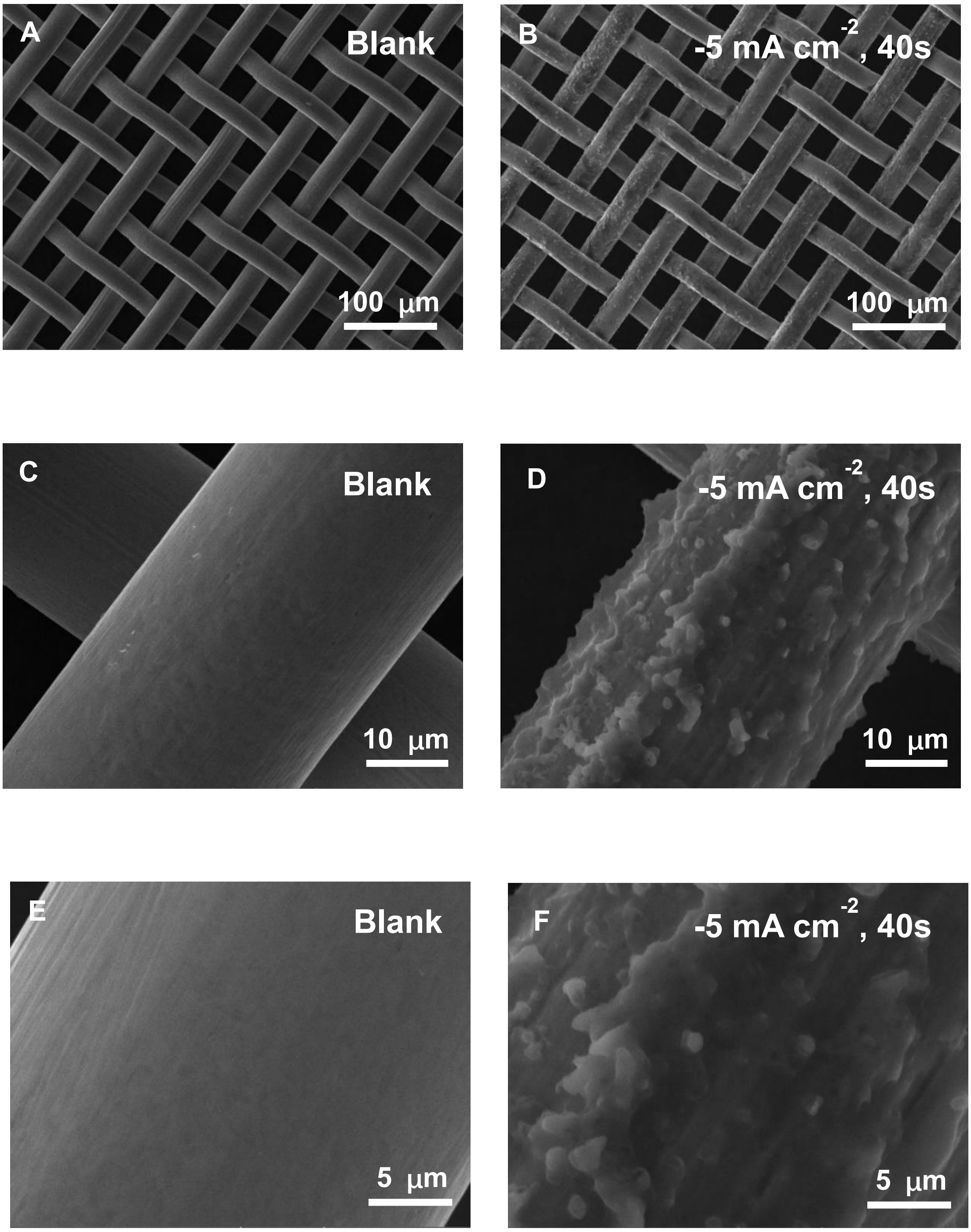

Figure S20. Related to STAR Methods. Scanning electron microscopy (SEM) images of stainless-steel cloths (SSCs) before (A, C, E) and after (B, D, F) lithium plating at current density of -5 mA cm^{-2} for 40 s in nitrogen saturated electrolyte containing 1 M $LiBF_4$.

Table S1. Literature data on performance of electrochemical nitrogen reduction reaction in non-aqueous electrolyte at ambient condition. **Related to Figure 4.**

Reference	Catalysts	Electrolyte	Type of electrolytic cell	Highest NH_3 rate ($\mu g\ s^{-1}\ cm^{-2}$)	FE to ammonia at highest rate %
**This work**	Stainless-steel cloth	1 M $LiBF_4$, 0.11 M EtOH in THF	Gas diffusion cell	0.410 ± 0.038	34.9 ± 1.7
(Lazouski *et al.*, 2019)	Cu foil	1 M $LiBF_4$, 0.2 M EtOH in THF	Two compartments cell	0.1339	15.2
(Akira et al., 1994)	Ag foil	0.2 M $LiBF_4$, 0.18 M EtOH in THF	Single compartment cell	0.0098	8.4
(Andersen et al., 2019)	Mo foil	0.2 M $LiBF_4$, 0.18 M EtOH in THF	Single compartment cell	0.0038	7.5
(Gao et al., 2020)	Au/CP	0.2 M $LiClO_4$, 1% EtOH in THF	Single compartment cell	0.0131	11
(Schwalbe et al., 2020)	Mo rod	0.5 M $LiClO_4$, 1 vol.% EtOH in THF	five-neck flask	0.0071	3.7
(Andersen et al., 2020)	Mo foil	0.3 M $LiClO_4$, 1 vol.% EtOH in THF	Single compartment cell	~0.0147	37.6
(Lee et al., 2018)	Ag-Au@ZIF	0.2M $LiCF_3SO_4$, ~1 % EtOH in THF	Single compartment cell	$1.7X10^{-4}$	18
(Tsuneto et al., 1993)	Ni	0.2 M $LiClO_4$, 1 vol.% EtOH in THF	Single compartment cell	0.0076	6.5
(Lazouski et al., 2020)	Stainless-steel cloth	1 M $LiBF_4$, 0.11 M EtOH in THF	Gas diffusion cell	0.516	35
(Zhou et al., 2017)	Stainless-steel cloth	100 ppm H_2O in [C4mpyr][eFAP]	H-type cell	$3.6X10^{-4}$	34
(Kim et al., 2016)	Ni foil	0.1 M LiCl in EDA	Two-chambered electrolysis cell	$6.1X10^{-4}$	17.2
(Pappenfus et al., 2009)	Ni plate	0.2 M LiClO4, 0.18 M EtOH in EMITFSI	Single compartment cell	0.0013	3.8
(Suryanto et al., 2018)	$Fe@Fe_3O_4$	Mixture of FPEE and [C4mpyr][eFAP]	Single compartment cell	0.0004	32
(Suryanto et al., 2021)	copper disk	0.2 M $LiBF_4$ + 0.1 M [P6,6,6,14][eFAP]	Single compartment cell	~0.995	69

Table S2. Literature data on performance of electrochemical nitrogen reduction reaction in aqueous electrolyte at ambient condition. **Related to Figure 4.**

Reference	Catalysts	Electrolyte	Type of electrolytic cell	Highest NH_3 rate ($\mu g\ s^{-1}\ cm^{-2}$)	FE to ammonia at highest rate %
(Shi et al., 2017)	Au/TiO_2	0.1 M HCl	H-type cell	0.0059	8.11
(Lv et al., 2018)	$Bi_4V_2O_{11}/CeO_x$	0.1 M HCl	H-type cell	0.0129	10.16
(Li et al., 2019)	Bi nanosheet	0.1 M H_2SO_4	H-type cell	$7.1X10^{-4}$	10.46
(Ba et al., 2020)	Mo_2C	0.1 M Na_2SO_4	H-type cell	$9.3X10^{-4}$	40.2
(Wang et al., 2018a)	Ru nanoparticles	0.01 M HCl	Electrochemical cell sealed with a Suba	$1.5X10^{-4}$	0.9
(Huang, 2019)	PdCu	0.5M LiCl	H-type cell	0.0099	11.5
(Xu et al., 2020)	$PdH_{0.43}$	0.1 M phosphate buffer solution	H-type cell	0.0016	43.6
(Yang et al., 2018)	VN	Humidified Nafion 211	Gas diffusion cell (MEA configuration)	0.0018	30.6
(Lan and Tao, 2013)	Pt/C	Humidified Nafion 211	Gas diffusion cell (MEA configuration)	$5X10^{-4}$ @20 ℃	~0.015 @ 20℃
(Tao et al., 2019)	Ru Single-Atom	0.1 M HCl	H-type cell	0.0061	7.5
(Wang et al., 2018b)	Fe−N/C	0.1M KOH	H-type cell	0.0048	9.28
(Luo et al., 2019)	Mxene $Ti_3C_2T_x$	0.5 M Li_2SO_4 (PH=2)	H-type cell	0.0013	4.62
(Wu et al., 2019)	$Fe\text{-}TiO_2$	0.5 M $LiClO_4$	H-type cell	$7.1X10^{-4}$	25.6

Table S3. Water content of solutions quantified by via Karl-Fischer titration. **Related to STAR Methods.**

Solution	Water content (ppm)		
	First test	Second test	Third test
THF	12.2	10.1	11.3
EtOH	6.8	7.0	7.1
1 M $LiBF_4$ in THF	23.1	22.0	23.9
1 M $LiBF_4$ + 0.11 M EtOH in THF	23.9	24.2	23.5
